# Discovery of a Novel DNMT1 Inhibitor with Improved Efficacy in Treating β‐Thalassemia

**DOI:** 10.1002/advs.202513469

**Published:** 2025-12-05

**Authors:** Yijie Shen, Jiale Wei, Shibing Tang, Dongliang Wu, Liangyi Zong, Shuyuan Ma, Qing Xiong, Ruijie Gong, Siyuan Xu, Chuxuan Peng, Qin Feng, Songchen Liu, Qitong Liu, Yuhua Ye, Quan Zhao, Cheng Luo, Peng Huang, Zhihai Li, Xiangqian Kong, Xianjiang Lan

**Affiliations:** ^1^ Department of Systems Biology for Medicine, School of Basic Medical Sciences, Department of Liver Surgery and Transplantation, Liver Cancer Institute Zhongshan Hospital Fudan University 130 Dong'An Road Shanghai 200032 China; ^2^ School of Pharmaceutical Science and Technology, Hangzhou Institute for Advanced Study University of Chinese Academy of Sciences No.1 Xiangshan Road Hangzhou 310024 China; ^3^ Institute of Drug Discovery China‐New Zealand Joint Laboratory on Biomedicine and Health Guangdong Provincial Key Laboratory of Stem Cell and Regenerative Medicine Guangzhou Institutes of Biomedicine and Health Chinese Academy of Sciences 190 Kaiyuan Avenue Guangzhou 510530 China; ^4^ The State Key Laboratory of Pharmaceutical Biotechnology, School of Life Sciences Nanjing University Nanjing 210023 China; ^5^ GMU‐GIBH Joint School of Life Sciences, The Guangdong‐Hong Kong‐Macao Joint Laboratory for Cell Fate Regulation and Diseases Guangzhou Medical University Guangzhou 511436 China; ^6^ Department of Hepatobiliary Surgery and Transplantation, Liver Cancer Institute, Zhongshan Hospital, Institutes of Biomedical Sciences, Key Laboratory of Carcinogenesis and Cancer Invasion of Ministry of Education, Key Laboratory of Medical Epigenetics and Metabolism Zhongshan Hospital Fudan University Shanghai 200032 China; ^7^ Innovation Center for Diagnostics and Treatment of Thalassemia, Nanfang Hospital, Southern Medical University, Guangzhou, Department of Medical Genetics, School of Basic Medical Sciences Southern Medical University Guangzhou Guangdong 510515 China; ^8^ State Key Laboratory of Drug Research, Shanghai Institute of Materia Medica Chinese Academy of Sciences 555 Zuchongzhi Road Shanghai 201203 China

**Keywords:** β‐thalassemia, DNA methylation, epigenetics, fetal hemoglobin (HbF), non‐nucleoside DNMT1 inhibitor

## Abstract

β‐thalassemia is a recessively inherited blood disorder affecting millions worldwide. Pharmacological induction of fetal hemoglobin (HbF) is an effective therapeutic strategy, yet existing DNA methyltransferase (DNMT) inhibitors, although effective HbF inducers, currently are not approved for β‐thalassemia treatment. Here, we report that DMT207, a novel non‐nucleoside DNMT1 inhibitor, robustly reactivates HbF in HUDEP‐2 cells and adult primary erythroblasts with minimal toxicity. In a mouse model of β‐thalassemia, DMT207 effectively elevates the levels of mouse fetal‐ and embryonic‐type hemoglobin, promotes the maturation of erythroid cells, and alleviates the splenomegaly. Further multi‐omics analyses expose γ‐globin as one of the most sensitive genes with promoter demethylation and transcriptional activation following DMT207 treatment. Mechanistically, DMT207 traps DNMT1 into a catalytically inactive conformation and concurrently enhances its interaction with UHRF1, which partially contributes to DNMT1 degradation. These findings highlight the therapeutic potential of DMT207 for β‐thalassemia and support its further preclinical development.

## Graphical abstract

**Context of Research**: β‐thalassemia affects millions worldwide. DNMT inhibitors are effective HbF‐inducers that benefit patients with β‐thalassemia. Existing DNMT inhibitors are not approved for β‐thalassemia treatment due to dose‐limiting toxicity.

**What We Find**: DMT207 traps DNMT1 into helix‐kinked inactive conformation and enhances its interaction with UHRF1. DMT207 potently induces HbF in vitro and in vivo, alleviating β‐thalassemia‐associated symptoms in mice with well tolerability. Multi‐omics analyses reveal *HBG1/2* as highly responsive targets, while global gene expression remains stable, suggesting specificity and safety.

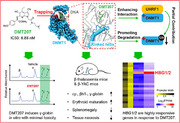

## 1 Introduction

β‐thalassemia is a monogenic, recessively inherited disease, caused by various mutations in the *HBB* gene, which lead to reduced or abolished synthesis of β‐globin chains in erythroid cells.^[^ ^1^ ^]^ These mutations disrupt the α/β‐globin balance, hindering erythroid maturation and survival, ultimately causing chronic anemia and a compromised quality of life.^[^ ^2^ ^]^ Curative treatments for transfusion‐dependent β‐thalassemia include hematopoietic stem cell transplantation and gene therapy, which either produce normal red blood cells (RBCs) or reactivate γ‐globin to restore the balance.^[^ ^3^, ^4^ ^]^ However, access to these interventions remains limited, particularly in low‐income countries, due to the substantial demand for medical resources.^[^ ^5^ ^]^ Therefore, there is an urgent need to develop more accessible therapeutic strategies, including small molecule drugs and biologics, to reduce the transfusion burden and alleviate anemia‐associated complications in patients with β‐thalassemia.

To date, several small molecule drugs and biologics have been approved for the treatment of β‐globinopathies, they can broadly be categorized into two purposes: reactivating fetal hemoglobin (HbF) or enhancing erythroid maturation.^[^ ^6^, ^7^, ^8^, ^9^, ^10^, ^11^ ^]^ Specifically, hydroxyurea, the only FDA‐approved HbF‐inducing agent for sickle cell disease (SCD), also provides some clinical benefits for a subset of patients with β‐thalassemia.^[^ ^12^ ^]^ Nonetheless, concerns regarding myelosuppression, response heterogeneity, and the need for continuous monitoring underscore the demand for new, safer HbF‐inducing agents.^[^ ^13^, ^14^ ^]^

A variety of epigenetic modulators have shown their efficacy in inducing HbF in preclinical models.^[^ ^15^, ^16^, ^17^, ^18^, ^19^ ^]^ However, only a few have been advanced into clinical trials.^[^ ^20^, ^21^, ^22^ ^]^ Among these, the nucleoside analog hypomethylating agents (HMAs), decitabine (DAC) and 5‐azacytidine (AZA), which target multiple DNA methyltransferases (DNMTs), effectively elevate HbF levels by inducing DNA hypomethylation at the *HBG1/2* promoters.^[^ ^19^, ^23^, ^24^ ^]^ This, in turn, alleviates hemolysis, and promotes erythroid maturation in patients.^[^ ^21^, ^22^ ^]^ However, these nucleoside analogs cause highly toxic DNA‐DNMTs crosslinks and genome‐wide DNA damage.^[^ ^25^, ^26^, ^27^, ^28^ ^]^

To address these limitations, significant efforts have been made on developing selective non‐nucleoside inhibitors of DNMT1, which is responsible for maintaining genome‐wide DNA methylation.^[^ ^29^, ^30^ ^]^ Recent studies have identified a class of dicyanopyridine‐containing DNMT1 inhibitors. These inhibitors bind both DNMT1 and hemi‐methylated DNA (hemi‐DNA), trapping DNMT1 into a helix‐kinked conformation.^[^ ^31^, ^32^ ^]^ Notably, the representative compound GSK3484862 promotes DNMT1 degradation in several cellular models.^[^ ^33^ ^]^ Furthermore, its structural racemate, GSK3482364 (GSK364), has been evaluated in human erythroid cells and in a transgenic SCD mouse model for HbF reactivation.^[^ ^34^ ^]^ However, it remains unclear how GSK364 affects the global DNA methylation patterns, transcriptional and proteomic profiles. Additionally, the mechanistic link between inhibitor‐induced conformational changes and protein degradation, as well as its functional implications in HbF reactivation, is largely uncharacterized. Moreover, the development of DNMT1 inhibitors with novel chemical structures may further improve selectivity, pharmacokinetic properties, and safety profiles, offering sustainable therapeutic benefits for patients with SCD and β‐thalassemia.

Here we report DMT207, a novel DNMT1 inhibitor with a 7‐azaindole skeleton, identified through a scaffold‐hopping study based on the GSK series compounds (manuscript in preparation). DMT207 exhibits markedly improved HbF‐inducing activity and minimal cytotoxicity in human umbilical cord blood‐derived erythroid progenitor (HUDEP)‐2 cells and adult primary erythroblasts, outperforming previously reported DNMT inhibitors. Furthermore, DMT207 induces fetal‐ and embryonic‐type hemoglobin in vivo and alleviates β‐thalassemia‐associated symptoms in model mice. Multi‐omics analyses reveal that, under an optimized dosing regimen, *HBG1/2* are among the most sensitive genes with promoter demethylation and exhibit robust transcriptional upregulation in response to DMT207 treatment. This effect is at least partially mediated by enhanced interactions between the conformationally‐kinked DNMT1 and UHRF1 (plant homeodomain and RING finger domains 1), which promotes DNMT1 degradation. Collectively, these findings suggest that DMT207 is a promising small molecule candidate for benefiting patients with β‐thalassemia.

## 2 Results

### 2.1 DMT207 Exhibits Superior HbF‐Inducing Capability Compared to Other DNMT1 Inhibitors

**Figure 1:**
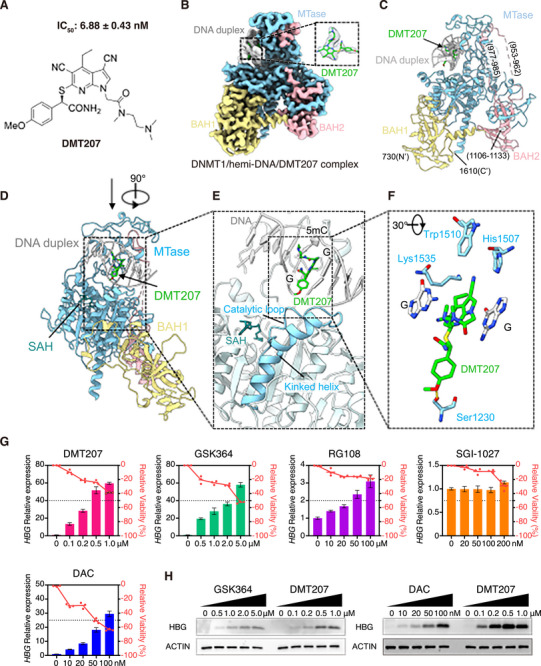
Cryo‐EM structure of the DNMT1/hemi‐DNA/DMT207 complex. A) Chemical structure of DMT207. The IC_50_ value is represented as the mean ± SD (n = 3). The IC_50_ of compound GSK3482364 is 2.84 ± 0.77 µm. B) Cryo‐EM density of DNMT1/hemi‐DNA/DMT207 ternary complex. The density of ligand DMT207 is highlighted. The hemi‐DNA, SAH and DMT207 are colored in light gray, cyan and green, respectively. DNMT1 is colored by domain: BAH1, khaki; BAH2, pink; MTase, blue. C,D), Cartoon representations of the DNMT1/hemi‐DNA/DMT207 complex in front (C) and side views (D). E) Close‐up view of boxed region in (D), showing the binding region of DMT207. The catalytic loop and DNA recognition helix with a kinked configuration are highlighted. F) Magnified view of boxed region in (E), revealing the detailed interactions among DMT207, hemi‐DNA and DNMT1. DMT207 inserts the DNA groove by forming *π*–*π* stacking with DNA bases. Besides, DMT207 forms hydrogen bonds with Ser1230 and van der Waals interactions with His1507, Trp1510 and Lys1535. For clarity, only the two guanine bases across two complementary DNA strands and key interacting residues with DMT207 are displayed. G) Relative expression of *HBG* (left y‐axis; bar plot; normalized to GAPDH) and relative viability (right y‐axis, red, line graph) in HUDEP‐2 cells after drug treatment. Results are shown as mean ± SD (n = 3). H) Western blots of γ‐globin in HUDEP‐2 cells after drug treatment.

To identify novel non‐nucleoside DNMT1 inhibitors, we conducted a scaffold‐hopping study based on the crystallographic structures of GSK series compounds in complex with DNMT1,^[^ ^31^, ^32^ ^]^ followed by medicinal chemistry optimization aimed at enhancing DNMT1 inhibitory activity (manuscript in preparation). This effort led to the discovery of a series of 7‐azaindole‐containing DNMT1 inhibitors, among which DMT207 showed over 400‐fold greater DNMT1 inhibition (IC_50_ = 6.88 ± 0.43 nM) than GSK364 (IC_50_ = 2.84 ± 0.77 µm), a previously reported HbF‐inducing compound^[^ ^34^ ^]^ (**Figure** **1**A). We then determined the cryo‐electron microscopy (cryo‐EM) structure of DNMT1/hemi‐DNA in complex with DMT207 at a global resolution of 2.85 Å (Figures 1B; S1A–D and Table S1, Supporting Information). The high‐resolution EM map clearly resolved the structure of the DNMT1 protein, including the BAH1, BAH2 and methyltransferase (MTase) domains, along with a 9‐bp DNA duplex containing centrally located hemi‐methylated CpG dyads, and the bound DMT207 (Figures 1B,C; S1E, Supporting Information). Similar to the binding modes of GSK inhibitors,^[^ ^31^, ^32^ ^]^ DMT207 displaced the catalytic loop (residues 1222–1235) of DNMT1 from penetrating into the hemi‐DNA for catalyzing methyl transfer by forming π‐π stacking, hydrogen bond and van der Waals interactions with both the DNA and protein (Figure 1D–F). Intriguingly, binding of DMT207 caused the DNA recognition helix (residues 1236–1259) to immediately follow the catalytic loop, adopting a kinked conformation rather than the extended, straight structure observed in the catalytically active DNMT1 state^[^ ^35^, ^36^ ^]^ (Figure 1D,E).

Next, we evaluated the effects of DMT207 and other DNMT1 inhibitors on γ‐globin expression in the human umbilical cord blood‐derived erythroid progenitor (HUDEP)‐2 cell line.^[^ ^37^ ^]^ Treatment with DMT207, GSK364 or DAC robustly upregulated γ‐globin mRNA and protein levels in a dose‐dependent manner while maintaining at least 50% cell viability (Figures 1G,H; S2A, Supporting Information). In contrast, two other reported non‐nucleoside DNMT inhibitors, RG108^[^ ^38^ ^]^ and SGI‐1027,^[^ ^39^ ^]^ were virtually incapable of doing so, likely due to their weak on‐target engagement with cellular DNMT1.^[^ ^31^ ^]^ Consistent with its higher DNMT1 inhibition potency in vitro, DMT207 outperformed GSK364 in activating γ‐globin expression at much lower concentrations (Figures 1G,H; S2B, Supporting Information). Importantly, there were no significant changes in *HBA*, *HBB* and *GATA‐1* mRNA levels (Figure S2C, Supporting Information), suggesting that DMT207 treatment did not overtly impair erythroid differentiation, an observation further supported by multi‐omics analyses (below, Figure 5). Although DAC, currently evaluated in a phase 2 clinical trial for SCD patients (NCT05405114), induced substantial γ‐globin expression at concentrations below 100 nm, its severe cytotoxicity limited dose escalation, preventing the attainment of the higher γ‐globin levels observed with DMT207 at concentrations above 200 nm (Figure 1G,H). This dose‐limiting toxicity likely arises from the formation of DNA‐DNMTs adducts, which are highly toxic DNA lesions induced by DAC upon incorporation into DNA and the subsequent covalent trapping of DNMTs.^[^ ^25^, ^40^ ^]^ Indeed, we observed dramatic accumulation of γ‐H2AX in DAC‐treated HUDEP‐2 cells, but not in those treated with DMT207 (Figure S2D, Supporting Information).

In summary, DMT207 represents a novel DNMT1 inhibitor with improved efficacy and tolerability for γ‐globin induction.

### 2.2 DMT207 Reactivates Fetal Hemoglobin Expression During Adult Erythropoiesis

**Figure 2:**
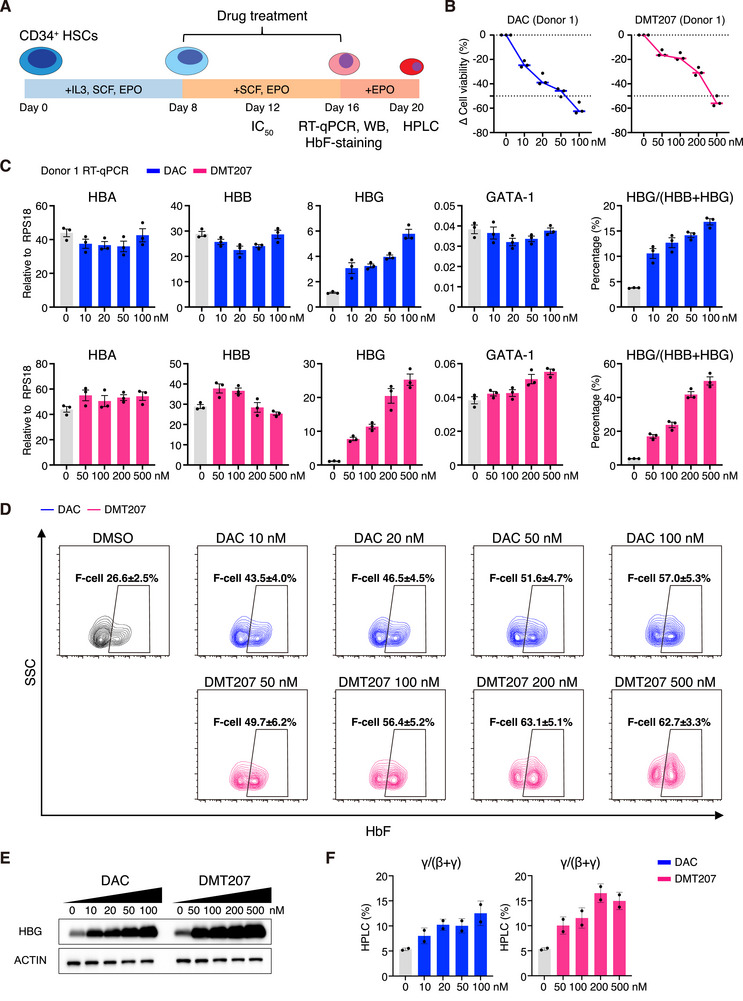
DMT207 elevates γ‐globin in adult primary erythroblasts (Donor 1). A) Experimental scheme of in vitro differentiation of adult primary erythroblasts. CD34^+^ HSCs were isolated from peripheral blood of healthy donors and cultured with indicated cytokines. Drug treatment last from day 8 to day 16. B) Relative cell viability of adult primary erythroblasts on day 16 (donor 1, n = 3). C) Relative expression of *HBA*, *HBB*, *HBG* and *GATA‐1* in adult primary erythroblasts from donor 1, treated with serial concentrations of DAC and DMT207 on day 16. The RPS18 gene was used for normalization. Results are shown as mean ± SD (n = 3). D) Flow cytometry analysis of HbF^+^ cells treated with serial concentrations of DAC and DMT207 on day 16. Results are shown as mean ± SD (n = 2). E) Western blots of adult primary erythroblasts, treated with serial concentrations of DAC and DMT207 on day 16. F) The ratio of γ‐globin to β‐like globin levels based on HPLC analysis in adult primary erythroblasts on day 20. Results are shown as mean ± SD (n = 2).

We further examined the HbF induction efficacy of DMT207 in adult primary erythroblasts. CD34^+^ cells were isolated from healthy donors and cultured through a three‐phase differentiation system (**Figure** **2**A). Cells were treated with compounds from day 8 to day 16, and the cytotoxicity (half maximal inhibitory concentration of cell viability, IC_50_) was determined on day 12 (Figure 2A). In 4‐day drug treatment assays, DMT207 exhibited lower cytotoxicity than DAC in primary erythroblasts (Figure S3A, Supporting Information), consistent with the observations in HUDEP‐2 cells (Figure S2A, Supporting Information). Based on these results, we performed 8‐day drug treatment assays, refreshing the compounds and medium every 4 days to encompass the major stages of erythroid differentiation and maturation. To preserve cells in a healthy state, we employed DAC at concentrations ranging from 10 to 100 nm and DMT207 at concentrations from 50 to 500 nm. On day 16, we examined cell viability and the mRNA levels of globin genes along with key erythroid regulators (Figures 2B,C; S3B–D, Supporting Information). Considering donor‐to‐donor variability, we performed the tests in two independent donors. Here we showed the results from donor 1 in Figure 2 and donor 2 in Figure S3 (Supporting Information).

At concentrations of 50–100 nm with ~50% cell viability loss, DAC induced a selective upregulation of γ‐globin, and increased the proportion of HbF‐expressing cells (F‐cells) to levels comparable to those achieved by equal doses of DMT207, without significant affecting erythroid differentiation marker GATA‐1 or other γ‐globin regulators including BCL11A, KLF1 and SOX6 (Figures 2C–E; S3B,D, Supporting Information). Notably, only ~20% of DMT207‐treated primary erythroblasts lost cell viability (Figures 2B; S3C, Supporting Information). This allowed DMT207 dosing to be increased to 200 nm, which further reduced DNA methylation at the *HBG1/2* promoters (Figure S3E, Supporting Information) and promoted stronger γ‐globin expression (Figures 2C–E; S3D, Supporting Information), while maintaining >60% cell viability (Figures 2B; S3C, Supporting Information). In line with these results, globin high‐performance liquid chromatography (HPLC) analysis and HbF immunofluorescence (IF) staining also confirmed potent γ‐globin induction upon DMT207 treatment (Figures 2F; S4A, Supporting Information).

To further evaluate whether DNMT1 inhibitors affect erythroid maturation and enucleation, we analyzed primary erythroblasts from 2 donors by Giemsa staining and flow analysis (Figures S4B–S6, Supporting Information). Notably, Giemsa staining results exhibited no marked differences in cell morphology between treated and untreated groups (Figure S4B, Supporting Information). Considering inter‐individual heterogeneity, neither compound significantly impaired erythroid maturation under tested conditions, as assessed by cell surface marker staining (BAND 3, CD49d, CD71 and CD235a; Figures S5 and S6, Supporting Information). While both compounds appeared to slightly perturb erythroid enucleation (non‐significant), as indicated by a raised cell population stained with a nucleic acid dye Syto 16 (Figure S7A, Supporting Information). This may be a common phenomenon observed for epigenetic modulators.^[^ ^41^, ^42^, ^43^ ^]^ Last, DAC triggered the accumulation of γ‐H2AX at high concentrations, and this was not observed in DMT207‐treated samples (Figure S7B, Supporting Information).

Collectively, these data demonstrate the greater potential of DMT207 over DAC in reactivating HbF along with better cellular tolerance.

### 2.3 DMT207 Elevates γ‐Globin Expression in Primary Erythroblasts from β‐Thalassemia Patients

Next, we investigated whether transient DNMT1 inhibition by DMT207 could lead to prolonged HbF de‐repression, similar to the epigenetic memory effect observed in pharmacological inhibition of the EZH2/PRC2 activity.^[^ ^44^ ^]^ To this end, HUDEP‐2 cells were treated with 200 nm of DMT207 for 4 days, followed by the induction of differentiation in the absence of the compound (Figure S8A, Supporting Information, regimen 1). Alternatively, cells were cultured for another 4 days without the compound before differentiation (Figure S8A, Supporting Information, regimen 2). For comparison, we included hydroxyurea (Hu), an FDA approved HbF‐inducing agent, following the same two regimens. Notably, the dramatic activation and promoter demethylation of γ‐globin persisted in both the 4‐day and 8‐day culture conditions after DMT207 removal (Figure S8B,C, Supporting Information). This was not the case for Hu, whose effects required its continuous presence. These results collectively suggest that transient DMT207 treatment is sufficient to induce durable HbF expression.

**Figure 3:**
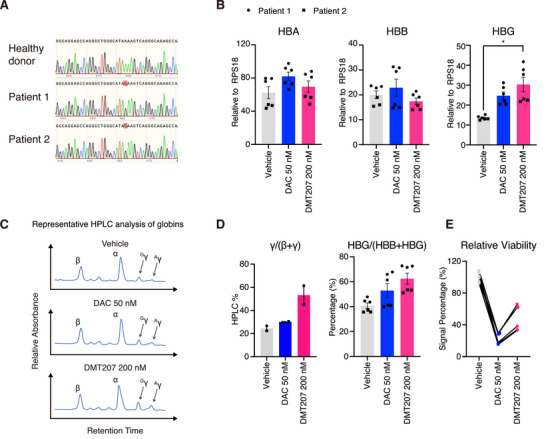
DMT207 elevates γ‐globin expression in CD34^+^ HSPCs from homozygous −28 A>G β‐thalassemia donors. A) Sanger sequencing of the *HBB* promoter. Two β‐thalassemia donors carry the homozygous −28 A>G mutation. B) Relative expression of *HBA*, *HBB*, *HBG* in CD34^+^ HSPCs from β‐thalassemia donors treated with DAC and DMT207 on day 16. Results are shown as mean ± SD (2 donors, for each donor, n = 3, ^*^ *p* < 0.05, statistical analysis was performed with Student's t‐test in the GraphPad Prism 10 software). C) Representative HPLC analysis of α‐ β‐ and γ‐globin levels on day 20. D) The ratio of γ‐globin to β‐like globin based on HPLC and RT‐qPCR analysis. Results are shown as mean ± SD (n = 2). E) Relative cell viability of erythroid cells on day 16 (2 donors, for each donor, n = 3).

Given that HbF induction confers clinical benefits to patients with β‐thalassemia,^[^ ^2^ ^]^ we thus evaluated the effects of DMT207 and DAC in primary erythroblasts derived from β‐thalassemia donors (See genotypes in **Figure** **3**A; previously reported in Orkin SH et al.^[^ ^45^ ^]^). Considering that the erythroid cells from β‐thalassemia patients are more fragile, here we employed an intermittent treatment strategy, in which compounds were applied only between day 8 to day 12 (See the three‐phase differentiation system in Figure 2A). Cells were treated with either vehicle, DAC (50 nm) or DMT207 (200 nm), considering the balance of efficacy and toxicity. On day 16, we determined the expression of globin by RT‐qPCR and HPLC (Figure 3B,C). Consistent with the results in primary erythroblasts from healthy donors, DMT207 specifically induced the expression of γ‐globin in both mRNA and protein levels, and maintained higher cell viability (Figure 3B–E).

Taken together, these data indicate that DMT207 represents a more effective and better‐tolerated therapeutic strategy than DAC in treating β‐globin disorders.

### 2.4 DMT207 Induces Fetal‐Type Hemoglobin Expression and Ameliorates β‐Thalassemia‐Associated Symptoms in Mice

**Figure 4:**
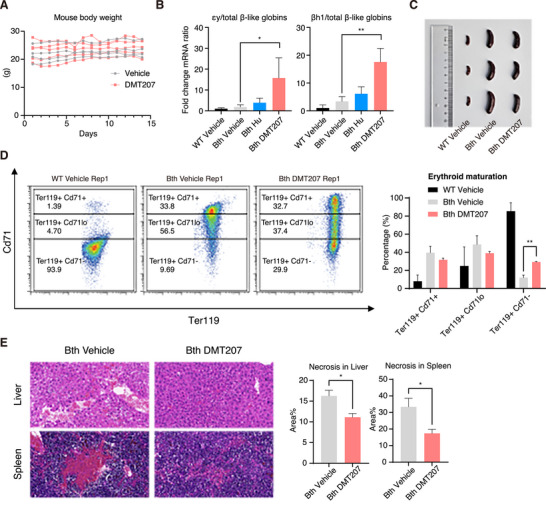
DMT207 induces εy‐ and βh1‐globin in β‐thalassemia mice. A) Mouse body weight of Bth‐vehicle and Bth‐DMT207 mice. B) RT‐qPCR quantification of εy‐ and βh1‐globin mRNA relative to total β‐like globin mRNA levels in bone marrow (normalized to mActin). Results are shown as mean ± SD (n = 5, ^*^ *p* < 0.05, ^**^ *p* < 0.01, statistical analysis was performed with Student's t‐test in the GraphPad Prism 10 software). C) Representative spleen picture of WT‐vehicle, Bth‐vehicle and Bth‐DMT207 mice at end of the experiments. D) Representative flow analysis of peripheral blood erythroid cells stained with cell differentiation markers Cd71 and Ter119. Results are shown as mean ± SD (n = 4, ^**^ *p* < 0.01, statistical analysis was performed with Student's t‐test in the GraphPad Prism 10 software). E) Hematoxylin and eosin (H&E) staining of liver and spleen sections from vehicle and DMT207 treated Bth mice with semiquantification of necrotic area analyzed by the ImageJ software. Results are shown as mean ± SD (n = 3, ^*^ *p* < 0.05, statistical analysis was performed with Student's t‐test in the GraphPad Prism 10 software).

To further evaluate the therapeutic potential of DMT207, we employed a mouse model of β‐thalassemia (C57BL/6 *^Hbbth−4/Hbb+^*, hereafter referred as Bth), in which a single allele of two murine adult β‐globin genes is replaced with the human βIVS‐2‐654 mutant allele.^[^ ^46^ ^]^ DMT207 (20, 50 or 100 mg kg^−1^), Hydroxyurea (Hu, 100 mg kg^−1^; an FDA‐approved fetal hemoglobin inducer) or vehicle was administered intraperitoneally to 6‐ to 8‐week‐old mice (n = 5) every weekday for 2 weeks (Figure S9A, Supporting Information). At the end of treatment, mice were sacrificed and subjected to downstream analyses. Of note, mouse weight was not significantly affected at the 50 mg kg^−1^ dose (**Figures** **4**A; S9B, Supporting Information), and this dosage effectively induced murine fetal‐ and embryonic‐type hemoglobin in bone marrow, comparable to the 100 mg kg^−1^ dose, implying a safe and effective therapeutic window (Figure S9C, Supporting Information). Importantly, DMT207 exhibited a stronger induction effect than Hu (Figure 4B). Moreover, splenomegaly, a common complication of β‐thalassemia (Figure 4C), was apparently mitigated following DMT207 treatment (Figure 4C).

Meanwhile, we assessed the peripheral blood parameters of mice, including erythroid maturation and hematological indices. Flow cytometry analysis using two erythroid differentiation markers Cd71 and Ter119 showed that there were only ~12% of all erythroid cells reached the Ter119⁺ Cd71^−^ stage, displaying impaired erythroid maturation in Bth mice, compared to wild‐type (WT) controls (Figures 4D; S9D, Supporting Information). In contrast, DMT207 treatment markedly raised this proportion to approximately one‐third (Figures 4D; S9D, Supporting Information), accompanied by minimal hematotoxicity (Table S2, Supporting Information). Importantly, the plateletcrit (PCT) of Bth mice partially restored to the normal range of WT mice, representing a biologically meaningful improvement (Table S2, Supporting Information). Additionally, tissue necrosis, another known pathological feature found in β‐thalassemia patients, was also observed in this mouse model, as shown by H&E staining on liver and spleen specimens (Figure 4E). Notably, DMT207 treatment significantly alleviated tissue damage in these organs (Figure 4E).

Finally, by employing the β‐YAC mouse model (n = 4), which contains the human β‐globin locus (*HBB* and *HBG*),^[^ ^47^ ^]^ we validated that DMT207 induced γ‐globin expression in vivo, alongside elevated murine fetal‐ and embryonic‐type hemoglobin (Figure S9E, Supporting Information).

Taken together, these in vivo findings demonstrate that DMT207 induces fetal‐type hemoglobin expression, mitigates key pathological features of β‐thalassemia, and is well tolerated in mice.

### 2.5 Multi‐Omics Analyses Reveal γ‐Globin as a Highly Sensitive Effector Gene Following DMT207 Treatment

**Figure 5:**
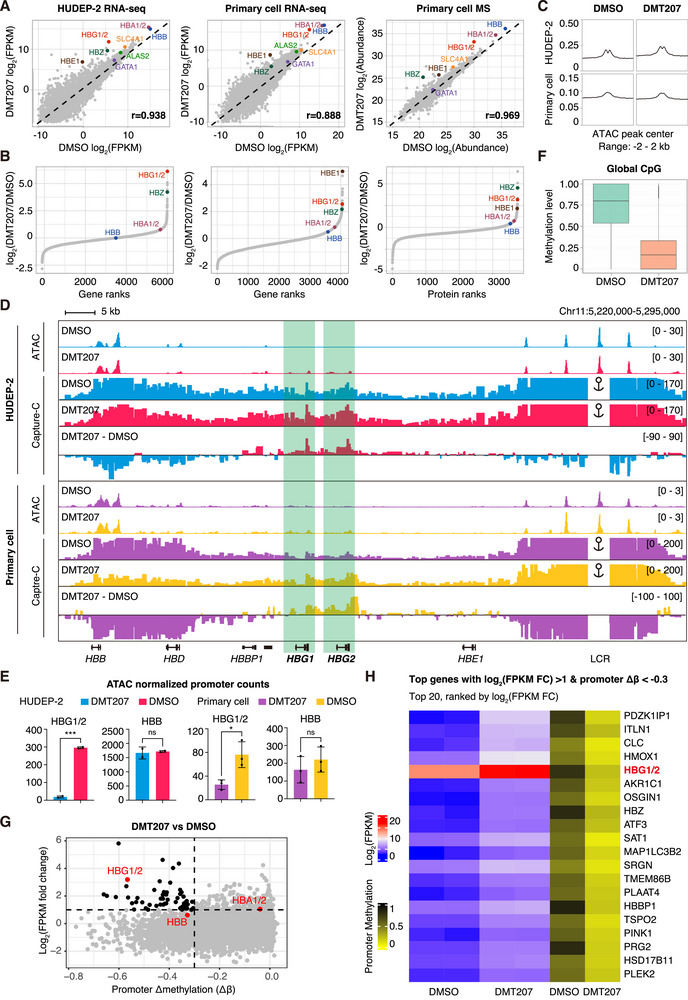
Multi‐omics analyses of global effects of DMT207. A) Scatter plots of RNA‐seq analysis in HUDEP‐2 cells and adult primary erythroblasts (DMT207 vs vehicle, left and middle). Scatter plot of mass‐spectrometry analysis in adult primary erythroblasts (DMT207 vs vehicle, right). B) Gene and protein ranks from RNA‐seq and mass‐spectrometry analysis in HUDEP‐2 cells and adult primary erythroblasts (DMT207 vs vehicle). C) Chromatin accessibility profiles of ATAC‐seq analysis in HUDEP‐2 cells and adult primary erythroblasts (DMT207 vs vehicle). D) Representative ATAC‐seq and Capture‐C tracks of the β‐globin locus in HUDEP‐2 cells and adult primary erythroblasts. E) Quantification of normalized ATAC‐seq reads enrichment at the *HBG1*/*2* and *HBB* promoters in HUDEP‐2 cells and adult primary erythroblasts. Results are shown as mean ± SD (n = 2 for HUDEP‐2 cells, n = 3 for adult primary erythroblasts; ^*^ *p* < 0.05, ^***^ *p* < 0.001, ns, not significant, statistical analysis was performed with Student's t‐test in the GraphPad Prism 10 software). F) Boxplot of global CpG methylation levels in adult primary erythroblasts, with n = 28081492 CpG sites detected for each group (DMSO & DMT207). G) Scatter plot of gene expression log_2_(FPKM fold change) vs promoter Δmethylation (Δβ) (DMT207 vs DMSO). H) Integrated heatmap of gene expression and promoter methylation. Genes with log_2_(FPKM FC) >1 and promotor Δβ < −0.3 are shown (n = 58, top20, rank of FPKM fold change).

To understand how DMT207 induces γ‐globin expression without disrupting normal erythropoiesis, we examined the global effects of DMT207 on HUDEP‐2 cells and adult primary erythroblasts through integrated transcriptomic and proteomic analyses (**Figure** **5**A). At a carefully selected concentration of 200 nm, at which DMT207 effectively drives γ‐globin expression without causing obvious cytotoxicity, the global gene and protein expression profiles were well maintained, as evidenced by the strong correlation between treated and untreated groups (r ≈ 0.9; Figure 5A). Strikingly, *HBG1/2* was robustly upregulated at both the mRNA and protein levels, and emerged as one of the most sensitive genes in response to DMT207 treatment, while adult globin genes and erythroid differentiation markers remained largely unchanged (Figures 5A,B; S10A, Supporting Information).

We next performed ATAC‐seq (assay for transposase‐accessible chromatin using sequencing) to examine how DMT207 selectively affects the chromatin accessibility of *HBG1/2* promoters in HUDEP‐2 cells and adult primary erythroblasts (Figure 5C,D). Consistent with the transcriptomic and proteomic data, the profile of global chromatin accessibility was not significantly changed following DMT207 treatment at this dosage (Figure 5C). It is worth noting that at the β‐globin locus DMT207 significantly increased the chromatin accessibility of the *HBG1/2* promoters by ATAC‐seq and enhanced the interactions between LCR (long control region) and *HBG1/*2 promoters by Capture‐C, rather than those of *HBB* (Figure 5D,E; Figure S10B, Supporting Information).

To further characterize the epigenetic mechanisms underlying this selective induction, whole‐genome bisulfite sequencing (WGBS) was performed to assess the global CpG methylation profiles in primary erythroblasts (Figure 5F). Notably, DMT207 treatment led to a global reduction in CpG methylation (Figure 5F). Given the well‐established transcriptional repressive role of promoter DNA methylation, we focused on promoter CpG methylation and integrated these data with transcriptomic profiles (Figures 5G,H; S10C, Supporting Information). Approximately one‐quarter of actively transcribed genes exhibited significant promoter demethylation (promoter Δ methylation (Δβ) < ‐0.3, n = 932; Figures 5G; S10C, Supporting Information), with *HBG1/2* ranked within the top 10% (Figure 5G; Table S3, Supporting Information). Importantly, only 58 genes exhibited concurrent promoter demethylation (Δβ < ‐0.3) and transcriptional upregulation (log_2_(FPKM) fold change > 1; Figure 5G). Among these, *HBG1/2* ranked among the top six genes in promoter demethylation and among the top five in transcriptional upregulation (Figure 5G,H; Table S4, Supporting Information). Moreover, re‐examination of the ATAC‐seq reads across promoter regions revealed that the *HBG1/2* promoters exhibited the most pronounced increase in chromatin accessibility among all actively transcribed genes (Figure S10D, Supporting Information).

Taken together, these data demonstrate that *HBG1/2* are among the most sensitive effector genes in response to DMT207 treatment in erythroid cells.

### 2.6 DMT207 Mediates DNMT1 Degradation Partially Through Enhancing its Interaction with UHRF1

**Figure 6:**
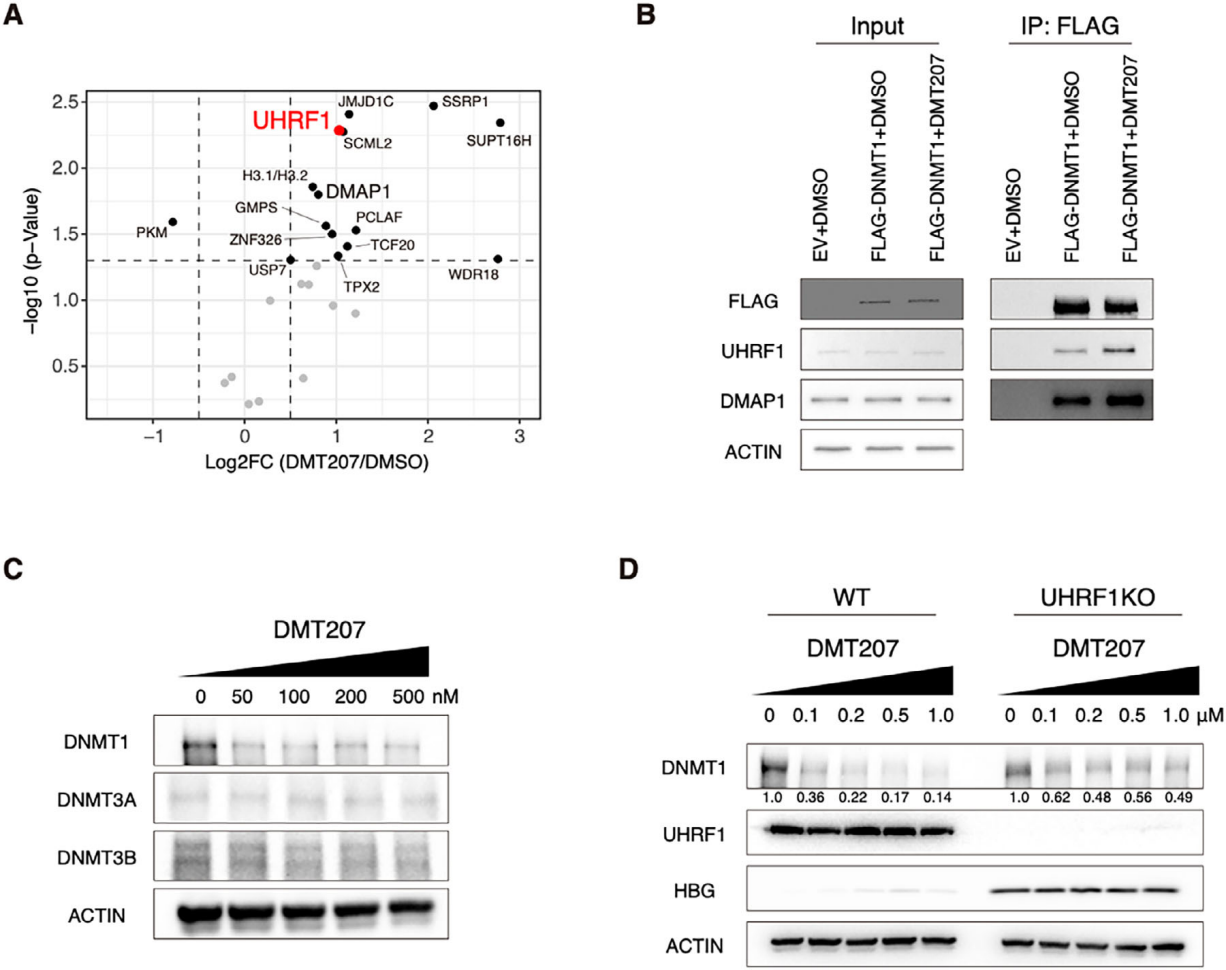
DMT207 mediates DNMT1 degradation by enhancing its interaction with UHRF1. A) Mass‐spectrometry analysis of FLAG‐tagged immunoprecipitates from HUDEP‐2 FLAG‐DNMT1 stable cells after 48 h treatment (DMSO vs DMT207). B) FLAG‐tagged IP‐WB of HUDEP‐2 FLAG‐DNMT1 cell lysates after 48 h treatment. C) Western blots of HUDEP‐2 cells treated with DMT207 for 2d. D) Western blots of HUDEP‐2‐Cas9 cells with non‐targeting or UHRF1 sgRNA treated with DMT207 for 2d.

Our cryo‐EM analysis uncovered that DMT207 stabilized DNMT1 into a helix‐kinked conformation (Figure 1D,E). We hypothesized that this conformational change may affect DNMT1 stability and its protein–protein interactions. To this end, we performed FLAG‐tagged immunoprecipitation coupled with mass‐spectrometry (IP‐MS) in FLAG‐DNMT1‐expressing HUDEP‐2 cells (**Figure** **6**A). Notably, UHRF1, known to recruit DNMT1 to replication forks during S phase,^[^ ^48^, ^49^ ^]^ was enriched in DMT207‐treated samples (Figure 6A; Table S5, Supporting Information), which was further validated by co‐immunoprecipitation (Co‐IP) assays in HUDEP‐2 cells and HEK293T cells (Figures 6B; S11A, Supporting Information). We also confirmed the IP‐MS results by blotting DMAP1, another known DNMT1 interactor (Figure 6B).

A recent study suggested that UHRF1 mediates DNMT1 degradation through its E3 ligase activity.^[^ ^50^ ^]^ Thus we speculated that DMT207 may promote DNMT1 degradation by enhancing its association with UHRF1. Indeed, DMT207 treatment led to a selective reduction in DNMT1 protein levels, which was also supported by our proteomic analysis (Figures 6C; S11B–D, Supporting Information). Importantly, UHRF1 depletion markedly, although not completely, blunted this process (Figure 6D). Together, these results indicate that in erythroid cells, DMT207 promotes DNMT1 degradation partially through enhancing its interaction with UHRF1.

## 3 Discussion

Reactivating fetal hemoglobin (HbF) in erythroid cells using small‐molecule inhibitors targeting epigenetic modulators is a promising strategy for the treatment of β‐globinopathies. However, only a few agents have been entered clinical evaluation, and their full therapeutic potential has been hindered by dose‐limiting toxicity, as exemplified by nucleoside analog‐based HMAs.^[^ ^25^, ^40^ ^]^ In this study, we identified DMT207, a novel non‐nucleoside DNMT1 inhibitor that robustly reactivated HbF expression both in vitro and in vivo, and ameliorated β‐thalassemia‐related symptoms in mice. Multi‐omics studies revealed that *HBG1/2* are among the most sensitive genes in response to DMT207 treatment in erythroid cells. Together, these findings demonstrate the superiorities of DMT207 with high HbF‐inducing capacity and low toxicity, and provide preclinical evidence supporting its potential application in β‐globin disorders.

Previous studies have shown that, upon loading hemi‐DNA, the DNA recognition helix of DNMT1 undergoes significant conformational changes, transitioning from a kinked to a straight configuration to facilitate DNA methylation.^[^ ^35^, ^36^, ^51^ ^]^ Our cryo‐EM analysis reveals that DMT207 stabilizes DNMT1 into a kinked conformation, which disrupts these dynamic transitions, resulting in reduced MTase activity and enhanced interactions with UHRF1.^[^ ^51^ ^]^ This mechanism is distinct from that of DAC, an HMA in clinical trials for β‐globin disorders,^[^ ^4^, ^21^ ^]^ which incorporates into DNA as a pseudo‐substrate to induce DNA‐DNMTs covalent adducts.^[^ ^52^ ^]^ These adducts are highly toxic lesions,^[^ ^25^, ^40^ ^]^ which results in dose‐limiting side effects and restricts the therapeutic window of DAC. In contrast, DMT207 exhibits a favorable safety profile, and this allows for more efficient HbF induction at higher concentrations with well overall tolerability.

Optimal drug dosing balances therapeutic efficacy against adverse effects.^[^ ^53^ ^]^ Both preclinical and clinal studies have highlighted the value of low‐dose DNA demethylating agents in oncology,^[^ ^54^ ^]^ which can reprogram the epigenome to elicit an antitumor “memory” response without immediate cytotoxicity,^[^ ^54^ ^]^ leading to improved patient outcomes compared to high doses.^[^ ^55^, ^56^ ^]^ Consistent with this paradigm, at a carefully selected concentration, DMT207 effectively induces HbF expression in primary erythroblasts from both healthy donors and β‐thalassemia patients, while maintaining cell viability. This also aligns with multi‐omics studies demonstrating substantial, selective promoter demethylation, increased promoter chromatin accessibility and transcriptional induction of *HBG1/2* in DMT207‐treated erythroid cells, while largely preserving the global transcriptome and proteome. Nevertheless, it should be noted that only 2‐week‐dosing regimens of DMT207 were tested for in vivo experiments, which is sufficient to activate fetal‐ and embryonic‐type hemoglobin expression and alleviate β‐thalassemia‐associated complications with well overall tolerability. DMT207 administration did not affect red blood cell parameters considerably and reduced markers of hypercoagulability, such as PCT. However, the lack of increase in total hemoglobin levels suggests that the current treatment duration may not yet achieve the maximal therapeutic effects. Further studies are warranted to explore prolonged treatment strategies and evaluate long‐term improvement in anemia symptoms.

Concerns raised by other DNMT inhibitors were also assessed in this study, such as the broad activation of the β‐globin locus, causing the leaky expression of *HBE1*, which could form embryonic hemoglobin, and could theoretically alter oxygen affinity or erythroid physiology. In our study, the level of *HBE1* reactivation was quantitatively minimal, with protein abundance constituting only ∼0.06% of total β‐like globins, which is unlikely to elicit major effects, upon 200 nm of DMT207 treatment. Besides, the leaky expression of other lineage genes may cause cell fate alteration. Importantly, RNA‐seq analysis revealed no significant changes in key erythroid regulators such as GATA‐1, while RUNX1 was moderately downregulated (~0.5‐fold), and the myeloid transcription factor SPI1 (PU.1) remained very low expression levels (0.7 to 7 FPKM) compared to GATA‐1 (~120 FPKM). Align with these results, flow cytometry analysis showed that >90% of cells were CD235a⁺ in both control and drug‐treated groups (Figure S6, Supporting Information, day 16–20), indicating the preservation of erythroid identity. Another important consideration is that epigenetic modulators may interfere with erythroid enucleation, as reviewed by Ji et al.^[^ ^41^ ^]^ Histone deacetylases (HDACs) or DNMTs inhibition could hinder chromatin condensation,^[^ ^41^, ^42^, ^43^ ^]^ which is a necessary process needed for erythroid terminal differentiation. We observed a slightly delayed erythroid enucleation, as indicated by a raised Syto 16^+^ cell population in both DAC and DMT207 treated groups (Figure S7A, Supporting Information). However, it is worth noting that DAC interfered more with enucleation than that of DMT207 under comparable levels of HbF induction (Figure S7A, Supporting Information).

In summary, we have developed DMT207, a novel non‐nucleoside DNMT1 inhibitor, and provide preclinical evidence supporting its potential application as a safe and effective therapeutic agent for β‐globin disorders.

## 4 Experimental Section

### Primary Cells

Human primary CD34^+^ cells from healthy donors were purchased from Oribiotech (Cat. #FmPB015F‐C). For patients‐derived primary cells, written informed consent from all the participants and/or their family members was obtained as outlined by the protocol approved by the Medical Ethics Committee of the Nanfang Hospital, Southern Medical University, Guangzhou, China (Approval number: NFEC2019‐039), as previously described.^[^ ^57^ ^]^ The study was conducted in accordance with the Declaration of Helsinki. CD34^+^ cells were isolated employing CD34 MicroBead Kit UltraPure (Miltenyi Biotec, Cat. #130‐100‐453).

### Erythroid Cell Culture and Differentiation

HUDEP‐2 cells were cultured as previously described.^[^ ^37^ ^]^ Briefly, HUDEP‐2 cells were maintained in StemSpan Serum Free Medium (SFEM, StemCell Technologies, Cat. #09605) supplemented with 1 µg mL^−1^ doxycycline (Glpbio, Cat. #GC13456), 1 µm dexamethasone (Sigma, Cat. #D4902), 50 ng mL^−1^ recombinant human stem cell factor (rhSCF, R&D, Cat. #7466‐SC), 3 IU mL^−1^ recombinant human erythropoietin (rhEPO, T&L Biotechnology, Cat. #GMP‐TL636), and 1% penicillin/streptomycin (P/S, Gibco, Cat. #15140122). HUDEP‐2 cells were induced differentiation in Iscove's Modified Dulbecco's Medium (IMDM, Gibco, Cat. #12440053) supplemented with 1 µg mL^−1^ doxycycline, 50 ng mL^−1^ rhSCF, 3 IU mL^−1^ rhEPO, 10 µg mL^−1^ insulin (MCE, Cat. #HY‐P0035), 10 µg mL^−1^ heparin (Sigma, Cat. #H3149), 320 µg mL^−1^ holo‐transferrin (R&D, Cat. #2914‐HT), 5% fetal bovine serum (FBS, ExCell Bio, Cat. #FSP500) and 1% P/S. Cells were kept at a density of less than 1.0 million mL^−1^.

CD34^+^ cells were cultured as previously described.^[^ ^58^ ^]^ Briefly, CD34^+^ cells were cultured in three‐phase systems in IMDM supplemented with 3 IU mL^−1^ rhEPO, 10 µg mL^−1^ insulin, 10 µg mL^−1^ heparin, 5% human male AB serum (Sigma, Cat. #H4522), and 1% P/S. Phase I medium was supplemented with 1 ng mL^−1^ recombinant human IL‐3 (PeproTech, Cat. #200‐03), 100 ng mL^−1^ rhSCF and 200 µg mL^−1^ holo‐transferrin. Phase II medium was supplemented with 100 ng mL^−1^ rhSCF and 200 µg mL^−1^ holo‐transferrin. Phase III medium was supplemented with 800 µg mL^−1^ holo‐transferrin. CD34^+^ cells were cultured in phase I medium for 8 days, and transitioned into phase II medium for 8 days, and finally transitioned into phase III medium for 4 days. Samples were harvested on day 20.

### RT‐qPCR

RNA samples were harvested in TRIzol (Invitrogen, Cat. #15596018CN), and extracted using standard procedures. cDNA was prepared using PrimeScript RT reagent Kit (Takara, Cat. #RR037A). qPCR reactions were prepared with FastStart Universal SYBR Green Master (Roche, Cat. #4913914001) and analyzed using Applied Biosystems QuantStudio 6 Pro systems. Quantification was performed with the ΔΔCT method.

### Western Blots and Antibodies

Western blotting was performed using standard procedures. Primary antibodies: DNMT1 (1:1000, CST, Cat. #5032), DNMT3A (1:1000, Proteintech, Cat. #20954‐1‐AP), DNMT3B (1:1000, Proteintech, Cat. #26971‐1‐AP), HBG1/2 (1:2000, Novus, Cat. #NB110‐41084), β‐actin (1:10000, Proteintech Cat. #66009‐1‐Ig), H2AX (1:1000, Proteintech, Cat. #10856‐1‐AP), γ‐H2AX (1:1000, Proteintech, Cat. #29380‐1‐AP), UHRF1 (1:1000, Proteintech, Cat. #21402‐1‐AP). Secondary antibodies: anti‐rabbit (1:10000, Proteintech, Cat. #RGAR001), anti‐mouse (1:10000, Proteintech, Cat. # SA00001‐1).

### HbF and Cell Surface Marker Flow Analysis

The HbF staining was performed as previously described.^[^ ^58^ ^]^ Briefly, 1 million cells were fixed with 0.05% glutaraldehyde (Sigma, Cat. #49629) for 10 min, and then permeabilized with 0.1% Triton X100 (Sigma, Cat. #X100) for 5 min. Subsequently, cells were stained with AF647‐conjucted HbF antibody (Novus, Cat. #NB110‐41084, Abcam, Cat. #ab269823) for 30 min in the dark at room temperature. Cells were then washed and analyzed by Thermo Attune NxT cytometer and software at Fudan University.

The cell surface marker staining was performed employing CD71‐PE (Biolegend, Cat. # 334106), CD235a‐PE/cy7 (Biolegend, Cat. #306620), mCd71‐PE (Biolegend, Cat. #113807), Ter119‐PE/cy5 (Biolegend, Cat. #113807). Cells were stained and analyzed by Thermo Attune NxT cytometer and software at Fudan University.

### Mouse Experiments

All procedures were conducted in accordance with the Fudan University Policy on the Care, Welfare and Treatment of Laboratory Animals, and were reviewed by the Institutional Animal Care and Use Committee at Fudan University. Six‐ to eight‐week‐old C57BL/6 *^Hbbth−4/Hbb+^* mice^[^ ^46^ ^]^ or β‐YAC mice^[^ ^47^ ^]^ were grouped and treated with either vehicle (5% DMSO + 20% Kolliphor EL + 75% PBS) or DMT207 (50 mg kg^−1^) via intraperitoneal injection on each weekday for 2 weeks. At the end of dosing, mice were sacrificed and subjected to downstream analysis.

### Statistical Analysis

Results were presented as mean ± SD/SEM. Differences of statistical significance were calculated based on the Students’ t test using GraphPad Prism Version 10. P ≤ 0.05 was represented as “*”, P ≤ 0.01 was represented as “**” and P ≤ 0.001 was represented as “***”, respectively. For RT‐qPCR data processing, GAPDH, RPS18 or mActin was used for normalization.

### Ethics Approval Statement and Patient Consent Statement

All animal experimental procedures were conducted in accordance with the Fudan University Policy on the Care, Welfare and Treatment of Laboratory Animals, and were reviewed by the Institutional Animal Care and Use Committee at Fudan University (Approval number: DSF‐2024‐057). Written informed consent from all the participants and/or their family members was obtained as outlined by the protocol approved by the Medical Ethics Committee of the Nanfang Hospital, Southern Medical University, Guangzhou, China (Approval number: NFEC2019‐039)

## Conflict of Interest

The authors declare no competing interests.

## Author Contributions

Y.S., J.W., and S.T. contribute equally to this work. Y.S. performed the experiments, analyzed the data and wrote the manuscripts. S.T. synthesized the compounds. J.W. and S.M. performed protein preparation and in vitro inhibition assays. Z.L. and J.W. performed cryo‐EM experiments and analysis. D.W. and Q.Z. provided model mice, and helped perform the mouse experiments. R.G. and Q.F. helped perform the mouse experiments. Y. Y. provided CD34^+^ cells from patients and assisted data analysis. Q.X. and P.H. performed Capture‐C experiments and analyzed data. S.X. and L.Z. helped analyze the data. C.P., S.L., and Q.L. helped perform IP experiments. C.L., Z.L., X.K., and X.L. designed the experiments. Z.L., X.K., and X.L. revised the manuscripts.

## Supporting information



Supporting Information

Supporting Table

## Data Availability

All data needed to evaluate the conclusions in the paper are present in the paper and/or the Supplementary Materials. The atomic coordinate and cryo‐EM map of DNMT1/hemi‐DNA/DMT207 complex were deposited into the Protein Data Bank (PDB) and Electron Microscopy Data Bank (EMD) under the session codes PDB 9V5P and EMD‐64791. The NGS data generated in this work were available at GEO under accession number GSE298941, GSE298942 and GSE298943.
