# Supplementary material for: Discovery of a Novel DNMT1 Inhibitor with Improved Efficacy in Treating β‐Thalassemia
Source: Adv Sci (Weinh). 2025 Dec 5;13(11):e13469. doi: 10.1002/advs.202513469 (PMC12931175; doi:10.1002/advs.202513469)
Supplement: Supplementary file 1 — Supporting Information [file ADVS-13-e13469-s001.docx]

**Supplemental data for:**

**Discovery of a novel DNMT1 inhibitor with**

**improved efficacy in treating β-thalassemia**

**Authors**

Yijie Shen **^1 #^**, Jiale Wei **^2 #^**, Shibing Tang **^3 #^**, Dongliang Wu **^4^**, Liangyi Zong **^3^**, Shuyuan Ma **^2^**, Qing Xiong **^5^**, Ruijie Gong **^6^**, Siyuan Xu **^1^**, Chuxuan Peng **^1^**, Qin Feng **^1^**, Songchen Liu **^1^**, Qitong Liu **^1^**, Yuhua Ye **^7^**, Quan Zhao **^4^**, Cheng Luo **^2,8^**, Peng Huang **^5^**, Zhihai Li **^2 *^**, Xiangqian Kong **^3 *^** and Xianjiang Lan **^1 *^**

**Affiliations**

**^1^** Department of Systems Biology for Medicine, School of Basic Medical Sciences, Department of Liver Surgery and Transplantation, Liver Cancer Institute, Zhongshan Hospital, Fudan University, Shanghai, 200032, China.

**^2^** School of Pharmaceutical Science and Technology, Hangzhou Institute for Advanced Study, University of Chinese Academy of Sciences, Hangzhou, 310024, China.

**^3^** Institute of Drug Discovery, China-New Zealand Joint Laboratory on Biomedicine and Health, Guangdong Provincial Key Laboratory of Stem Cell and Regenerative Medicine, Guangzhou Institutes of Biomedicine and Health, Chinese Academy of Sciences, Guangzhou, 510530, China.

**^4^** The State Key Laboratory of Pharmaceutical Biotechnology, School of Life Sciences, Nanjing University, Nanjing, 210023, China.

**^5^** GMU-GIBH Joint School of Life Sciences, The Guangdong-Hong Kong-Macao Joint Laboratory for Cell Fate Regulation and Diseases, Guangzhou Medical University, Guangzhou, 511436, China.

**^6^** Department of Hepatobiliary Surgery and Transplantation, Liver Cancer Institute, Zhongshan Hospital, Institutes of Biomedical Sciences, Key Laboratory of Carcinogenesis and Cancer Invasion of Ministry of Education, Key Laboratory of Medical Epigenetics and Metabolism, Zhongshan Hospital, Fudan University, Shanghai, 200032, China.

**^7^** Innovation Center for Diagnostics and Treatment of Thalassemia, Nanfang Hospital, Southern Medical University, Guangzhou, Department of Medical Genetics, School of Basic Medical Sciences, Southern Medical University, Guangzhou, Guangdong, 510515, China.

**^8^** State Key Laboratory of Drug Research, Shanghai Institute of Materia Medica, Chinese Academy of Sciences, 555 Zuchongzhi Road, Shanghai, 201203, China.

**^#^ These authors contribute equally to this work.**

**^*^ Address correspondence to these authors.**

**Supplemental** **Methods**

**Protein Expression and Purification**

The full-length human DNMT1 gene was chemically synthesized with codon optimization (BEIJING Tsingke Biotech Co., Ltd). Truncated DNMT1 constructs (residues 330-1616 or 698-1616) were cloned into the pFastBac baculovirus expression vector, incorporating an N-terminal 6×His-FLAG tag and TEV protease site. The proteins were generated in Spodoptera frugiperda 9 (Sf9) cells. Insect cell cultures were grown in ESF 921 serum-free medium (Expression Systems) to a density of approximately 3 million cells per mL and infected with baculovirus for 72 hours at 27°C to induce protein expression.

Cell pellets were resuspended in lysis buffer (50 mM HEPES, pH 8.0, 500 mM NaCl, 20 mM imidazole, 1 mM TCEP) with 1% protease inhibitor cocktail. Cells were lysed using a high-pressure homogenizer and the lysate was then centrifuged at 42,000 × g for 2 hours at 4°C. The supernatant was incubated with Ni-NTA beads (Smart-Lifesciences Biotechnology Co., Ltd.) for 2 hours at 4°C. After binding, the beads were washed with 20 column volumes of wash buffer (50 mM HEPES, pH 8.0, 500 mM NaCl, 20 mM imidazole, 1 mM TCEP). DNMT1 was eluted with 3-4 column volumes of elution buffer (50 mM HEPES, pH 8.0, 500 mM NaCl, 1 M imidazole, 1 mM TCEP). The eluted protein was concentrated using a centrifugal filter unit (Millipore Corporation) and further purified by size-exclusion chromatography on a Superdex 200 Increase 10/300 GL column (Cytiva), equilibrated with 20 mM Tris-HCl (pH 7.4), 150 mM NaCl, and 1 mM TCEP.

***In Vitro* DNMT1 Enzymatic Activity Assay**

DNMT1 enzymatic activities were measured using the MTase-Glo™ Methyltransferase Assay Kit (Promega V7601) as previously described ^[1]^. Briefly, DNA methylation reactions were performed at 37^°^C for 1 hour with 10 μL mixture containing 150 nM DNMT1 (residues 330-1616), 5 μM S-adenosyl-l-methionine (SAM), 2 μM hemimethylated DNA (hemi-DNA), and inhibitors at the indicated concentrations in 50 mM Tris-HCl (pH 7.4) and 1 mM EDTA. The hemi-DNA oligonucleotide (forward: 5’-ACTTAMGGAAGG-3’; reverse: 5’-CCTTCCGTAAGT-3’; where M = 5mC) contains a single hemi-methylated CpG site ^[2]^. Following the reaction, MTase-Glo™ Reagent and Detection solutions were added to convert the methylation product, S-adenosyl homocysteine (SAH), into ATP, which was detected by a luciferase reaction. The luminescence was measured using a microplate reader (PerkinElmer). The dose-response curves and the calculate IC_50_ values were generated using GraphPad Prism v.8.0.1.

**Cryo-EM Grid Preparation and Data Collection**

For the cryo-EM studies, we incubated the DNMT1/hemi-DNA complex with 5-fold molar excess of DMT207. To prepare cryo-EM grids, 3.0 μL of the purified DNMT1 complex at 0.5 mg/mL was applied onto glow-discharged Quantifoil R1.2/1.3 300-mesh gold holey carbon grids. The grids were blotted under 100% humidity at 4 °C and then plunge-frozen in liquid ethane cooled by liquid nitrogen using a Vitrobot Mark IV (Thermo Fisher Scientific). The frozen grids were stored in liquid nitrogen for data acquisition. Movies were collected on a 300 kV Titan Krios (FEI) equipped with a Gatan K3 Summit direct electron detector and a Gatan energy filter operated with a slit width of 10 eV (GIF). SerialEM ^[3]^ was used to automatically acquire micrographs at a pixel size of 1.071 Å and defocus values ranging from -1.5 to -3 μm. A total of 9,972 movies were collected at a total dose of 72 e-/Å^2^ with a total of 40 frames per movie.

**Cryo-EM Data Process**

The movies of DNMT1/hemi-DNA/DMT207 complex were processed using CryoSPARC v4.4 ^[4]^. The motion correction for the movies and the estimation of contrast transfer function (CTF) for the corrected micrographs were using programs *Patch Motion Correction* and *Patch CTF Estimation*, respectively. 9,341 good micrographs were selected after discarding the ones with crystal ice contamination. About 480,541 particles were picked using *Blob Picker* from 500 selected micrographs with relatively high defocus level ranging from -2.5 to -3.0 μm. The particles were extracted and subjected to a round of 2D classification. 2D averages with different orientations were selected and used as templates for automatic particle picking using *Template Picker*. A total of 9,819,985 particles were extracted and processed with two rounds of 2D classifications, yielding 2,214,698 particles generated from 2D averages with high-resolution structural features. An initial model for 3D classifications was generated *de novo* from selected particles using *Ab-initio Reconstruction*. The three generating volumes were used as template for the heterogeneous refinement. After rounds of heterogeneous refinement, 338,318 particles from a 3D class with high quality structural feature were selected and subjected to a round of Non-uniform Refinement, yielding a reconstruction of DNMT1/hemi-DNA/DMT207 at a global resolution of 2.85 Å based on the gold-standard Fourier shell correlation (FSC) = 0.143 criterion ^[5]^. Local resolution was calculated using *Local Resolution Estimation*. To improve the quality of the maps, two half maps from the final refinement were provided without a mask as input to DeepEMhancer ^[6]^.

**Model Building and Refinement**

The model building of DNMT1/hemi-DNA/DMT207 was started by using the structure of DNMT1/hemi-DNA/GSL3685032 (PDB ID: 6X9K) ^[7]^ as an initial model. After docking the model onto the density map of DNMT1/hemi-DNA/DMT207 using Chimera ^[8]^, it was manually adjusted in Coot ^[9]^ and the refined against the density map using Phenix. The figures were prepared using Chimera and ChimeraX ^[10]^.

**Small Molecule Compounds**

RG108, SGI-1027, DAC and GSK3482364 were obtained commercially from MCE (Cat. #HY-13642, #HY-13962, #HY-A0004, and #HY-135146A respectively). DMT207 was synthesized from commercially available regents following the procedure reported in patent CN2024104035592 and characterized by mass spectrometry (API 2000 electrospray mass spectrometer) and proton nuclear magnetic resonance (^1^H NMR) spectroscopy (Bruker AV-500 MHz spectrometer). The characterization data are as follows: ^1^H NMR (500 MHz, DMSO-*d*_6_) *δ* 8.46 + 8.42 (2x s, 1H), 7.80 (br s, 1H), 7.55 + 7.28 (2x br s, 1H), 7.45 (d, *J* = 8.7 Hz, 2H), 6.94 + 6.90 (2x d, *J* = 8.7 Hz , 2H), 5.60 + 5.49 (2x s, 1H), 5.49–5.25 (m, 2H), 3.76 + 3.74 (2x s,3H), 3.74–3.69 + 3.58–3.55 + 3.21–3.18 (3x m, 2H), 3.18–3.14 (m, 2H), 3.11 + 2.93 (2x s, 3H), 2.58–2.52 + 2.50–2.44 + 2.36–2.32 (3x m, 2H), 2.26 + 2.13 (2x s, 6H), 1.33 (t, *J* = 7.4 Hz, 3H). MS (ESI) *m/z*: calcd for C_27_H_32_N_7_O_3_S [M + H]^+^, 534.2; found, 534.3.

**Cell Culture**

HEK293T cells were cultured in Dulbecco's Modified Eagle Medium (DMEM) with high glucose (Cytiva, Cat. #SH30243.01), supplemented with 5% FBS and 1% P/S.

**Plasmid Construction**

To prepare U6-driven sgRNA expression vector, the LRG2.1 plasmid (Addgene, Cat. #108098) was digested employing BsmBI-v2 (NEB, Cat. #R0739S), and ligated with annealed complementary oligonucleotides containing the sgRNA sequence using T4 DNA Ligase (NEB, Cat. #M0202S). To prepare FLAG-DNMT1 expression vector, the PSDM101 plasmid ^[11]^ was digested with MluI-HF^®^ (NEB, Cat. # R3198S) and NdeI (NEB, Cat. #R0111S), and was ligated with synthesized FLAG-DNMT1 sequence using T4 DNA Ligase. See Tab. S5 for the list of all primers used in this study.

**Lentiviral Production and Infection**

Lentivirus was produced employing HEK293T cells in DMEM. Cells were transfected with 10 μg lentiviral expression vector (LRG2.1 or PSDM101), 7.5 μg lentiviral packaging plasmid (psPAX2, Addgene, Cat. #12260) and 5 μg envelope expressing plasmid (pMD2.G, Addgene, Cat. #12259). Briefly, plasmids were mixed in 500 μL Opti-MEM I (Gibco, Cat. #31985070), and add additional pre-mixed 500 μL Opti-MEM I containing 80 μL of 1 mg/mL polyethylenimine (PEI, Polysciences, Cat. #23966). The whole mixture was vortexed and incubated for 15 minutes and then added to HEK293T cells with refreshed DMEM. Six hours later, the medium was refreshed to remove PEI, and virus was harvested on day 2.

HUDEP-2 cells were infected following the spin-infection protocol ^[12]^. Briefly, 0.5 million HUDEP-2 cells were grown in 1 mL medium, and were infected with 1 mL virus supplemented with 8 µg/ml polybrene (MCE, Cat. #HY-112735) and 10 mM HEPES (Gibco, Cat. #15630080). HUDEP-2 cells and virus were incubated and subjected to spin-infection at 2250 rpm for 1.5 hours at room temperature. After spin-infection, cells were transferred to fresh medium.

**Hemoglobin HPLC**

Hemoglobin HPLC analysis was performed as previously described ^[13, 14]^. Cells were lysed in MilliQ water, then cleared by centrifugation and filtered through 0.4 μm cellulose filters. Chromatography was conducted using a Waters µBondapak C₁₈ column (10 µm particle size). The two solvent systems were employed: Solvent A (80:15:5:0.1 (v/v) of sodium perchlorate (0.15 M): acetonitrile: methanol: 85% phosphoric acid), Solvent B: 20:75:5:0.1 (v/v) of sodium perchlorate (0.15 M): acetonitrile: methanol: 85% phosphoric acid). The column was initially equilibrated with a mixture of 34% Solvent A and 66% Solvent B. After sample loading, an isocratic elution was maintained for 10 minutes, followed by a linear gradient from 66% to 70% Solvent B over 80 minutes. Quantification of globin chains was based on the integrated area under each elution peak.

**Immunoprecipitation**

About 100 million cells were washed and lysed with buffer A (10 mM HEPES pH 7.5, 1.5 mM MgCl2, 10 mM KCl, 1 mM DTT, and 1 mM PMSF) for 30 min. Cell nuclei were collected and lysed in buffer C (20 mM Tris-HCl pH 7.9, 25% glycerol, 420 mM NaCl, 1.5 mM MgCl2, 0.1% NP-40, 0.2 mM EDTA, 1 mM DTT, and 1 mM PMSF) for 30 min with Benzonase nuclease supplemented. Then, the solution was diluted with equal volume of buffer A, and the supernatant was collected for further steps. FLAG-beads were purchased from Thermo Scientific (Cat. #A36797). The beads were added to supernatant to incubate over night at 4°C. On the next day, beads were washed with buffer 150 (20 mM Tris-HCl pH 7.9, 25% glycerol, 150 mM NaCl, 1.5 mM MgCl2, 0.1% NP-40, 0.2 mM EDTA, 1 mM DTT, and 1 mM PMSF) once, buffer 350 (20 mM Tris-HCl pH 7.9, 25% glycerol, 350 mM NaCl, 1.5 mM MgCl2, 0.1% NP-40, 0.2 mM EDTA, 1 mM DTT, and 1 mM PMSF) once, and another twice with buffer 150. After that, beads were eluted with FLAG peptide for 4 h at 4°C. Then the elute was subjected to SDS-gel analysis.

**Proteomics Analysis via nLC-MS/MS**

Trypsin (Thermo Scientific, Cat. #90057) was employed to digest proteins from gels. The peptides were then collected and subjected to Nanospray Fle Ion Sources (Thermo Scientifi), followed by tandem mass spectrometry (MS/MS) in Orbitrap Exploris 480 MS coupled with Ultra Performance Liquid Chromatography (UPLC). Subsequently, tandem mass spectra were extracted by Proteome Discoverer software (Thermo Scientific, version 3.0) and searched against the Human database. The acceptance criteria for identifications were the false discovery rate (FDR) should be less than 1% for peptides and proteins.

**RNA-seq**

mRNA was captured using poly-T beads (Vazyme, Cat. #N401), and then was reverse-transcribed into cDNA to construct sequencing libraries using VAHTS TruePrep^®^ RNA Library Prep Kit for Illumina following manufacturer’s instructions (Vazyme, Cat. #TR501). RNA-seq libraries were sequenced using the Illumina NovaSeq 6000 platform.

**ATAC-seq**

ATAC-seq experiments were performed using Hyperactive ATAC-Seq Library Prep Kit for Illumina following to manufacturer’s instructions (Vazyme, Cat. #TD711). All ATAC-seq libraries were sequenced using the Illumina NovaSeq 6000 platform.

**Capture-C**

Capture-C experiments were performed using NlaIII (NEB, Cat. #R0125L) digestion as previously described ^[15]^. The sequencing reads were processed using published scripts ^[16]^. Capture-C interactions of biological replicates were combined and normalized to total interactions.

**WGBS**

Whole genome bisulfite sequencing (WGBS) was performed as previously described. ^[17]^. Briefly, genomic DNA was extracted using DNeasy Blood & Tissue Kit (QIAGEN), and bisulfite-converted using Zymo-Seq WGBS Library Kit. Library was prepared using Zymo-Seq WGBS Library Kit, following manufacturer’s instructions. Libraries were sequenced using the Illumina NovaSeq 6000 platform to acquire 90 Gb data per sample. This corresponds to 30× coverage of the human genome.

**NGS Data Analysis**

For RNA-seq analysis, raw data files were trimmed and aligned to hg38 genomes using HISAT2 software ^[18]^ with default parameters. The outputs SAM files were then transformed into BAM format using SAMtools ^[19]^ and extracted with FPKM using Stringtie2 software ^[20]^. In downstream analyses, genes with FPKM ≤ 5 were filtered. RNA-seq data generated in this study can be accessed at GEO database (GSE298942).

For ATAC-seq analysis, raw data was trimmed and aligned to hg38 genomes using Bowtie2 software ^[21]^. Unmapped reads and PCR duplicates were removed using SAMtools ^[19]^. Then, Bigwig files and were generated and normalized using deepTools ^[22]^. Promoter peaks were called using MACS2 software ^[23]^. ATAC-seq data generated in this study can be accessed at GEO database (GSE298941).

For WGBS analysis, raw data was trimmed and aligned to C>T and G>A converted genomes using Bismark software ^[24]^, and then methylation levels of CpG islands were called using Bismark methylation extractor command lines ^[24]^. WGBS data generated in this study can be accessed at GEO database (GSE298943).

**References**

1. Horton, J.R., et al., *Structural characterization of dicyanopyridine containing DNMT1-selective, non-nucleoside inhibitors.* Structure, 2022. **30**(6): p. 793–802 e5.

2. Adam, S., et al., *DNA sequence-dependent activity and base flipping mechanisms of DNMT1 regulate genome-wide DNA methylation.* Nat Commun, 2020. **11**(1): p. 3723.

3. Mastronarde, D.N., *Automated electron microscope tomography using robust prediction of specimen movements.* J Struct Biol, 2005. **152**(1): p. 36–51.

4. Punjani, A., et al., *cryoSPARC: algorithms for rapid unsupervised cryo-EM structure determination.* Nat Methods, 2017. **14**(3): p. 290–296.

5. Rosenthal, P.B. and R. Henderson, *Optimal determination of particle orientation, absolute hand, and contrast loss in single-particle electron cryomicroscopy.* J Mol Biol, 2003. **333**(4): p. 721–45.

6. Sanchez-Garcia, R., et al., *DeepEMhancer: a deep learning solution for cryo-EM volume post-processing.* Commun Biol, 2021. **4**(1): p. 874.

7. Pappalardi, M.B., et al., *Discovery of a first-in-class reversible DNMT1-selective inhibitor with improved tolerability and efficacy in acute myeloid leukemia.* Nat Cancer, 2021. **2**(10): p. 1002–1017.

8. Pettersen, E.F., et al., *UCSF Chimera--a visualization system for exploratory research and analysis.* J Comput Chem, 2004. **25**(13): p. 1605–12.

9. Emsley, P. and K. Cowtan, *Coot: model-building tools for molecular graphics.* Acta Crystallogr D Biol Crystallogr, 2004. **60**(Pt 12 Pt 1): p. 2126–32.

10. Pettersen, E.F., et al., *UCSF ChimeraX: Structure visualization for researchers, educators, and developers.* Protein Sci, 2021. **30**(1): p. 70–82.

11. Chevalier, S.A., et al., *The transcription profile of Tax-3 is more similar to Tax-1 than Tax-2: insights into HTLV-3 potential leukemogenic properties.* PLoS One, 2012. **7**(7): p. e41003.

12. Grevet, J.D., et al., *Domain-focused CRISPR screen identifies HRI as a fetal hemoglobin regulator in human erythroid cells.* Science, 2018. **361**(6399): p. 285–290.

13. Zago, M.A. and L.J. Greene, *An HbF enrichment procedure for the HPLC analysis of gamma chains.* Clin Chim Acta, 1985. **148**(1): p. 39–46.

14. Shelton, J.B., J.R. Shelton, and W.A. Schroeder, *Preliminary experiments in the separation of globin chains by high performance liquid chromatography.* Hemoglobin, 1979. **3**(5): p. 353–8.

15. Huang, P., et al., *HIC2 controls developmental hemoglobin switching by repressing BCL11A transcription.* Nat Genet, 2022. **54**(9): p. 1417–1426.

16. Davies, J.O., et al., *Multiplexed analysis of chromosome conformation at vastly improved sensitivity.* Nat Methods, 2016. **13**(1): p. 74–80.

17. Lister, R., et al., *Human DNA methylomes at base resolution show widespread epigenomic differences.* Nature, 2009. **462**(7271): p. 315–22.

18. Kim, D., et al., *Graph-based genome alignment and genotyping with HISAT2 and HISAT-genotype.* Nat Biotechnol, 2019. **37**(8): p. 907–915.

19. Li, H., et al., *The Sequence Alignment/Map format and SAMtools.* Bioinformatics, 2009. **25**(16): p. 2078–9.

20. Kovaka, S., et al., *Transcriptome assembly from long-read RNA-seq alignments with StringTie2.* Genome Biol, 2019. **20**(1): p. 278.

21. Langmead, B. and S.L. Salzberg, *Fast gapped-read alignment with Bowtie 2.* Nat Methods, 2012. **9**(4): p. 357–9.

22. Ramirez, F., et al., *deepTools2: a next generation web server for deep-sequencing data analysis.* Nucleic Acids Res, 2016. **44**(W1): p. W160–5.

23. Zhang, Y., et al., *Model-based analysis of ChIP-Seq (MACS).* Genome Biol, 2008. **9**(9): p. R137.

24. Krueger, F. and S.R. Andrews, *Bismark: a flexible aligner and methylation caller for Bisulfite-Seq applications.* Bioinformatics, 2011. **27**(11): p. 1571–2.

**Supplemental Tables**

**Supplemental Table 1. Cryo-EM data collection, image processing, and refinement statistics**

|  | DNMT1/hemiDNA/DMT207  (EMD: 64791; PDB: 9V5P) |
| --- | --- |
| **Data collection and processing** |  |
| Voltage (kV) | 300 |
| Electron exposure (e–/Å2) | 72 |
| Defocus range (μm) | -1.0 to -3.0 |
| Pixel size (Å) | 1.071 |
| Symmetry imposed | C1 |
| Initial particle images (no.) | 9,819,985 |
| Final particle images (no.) | 338,318 |
| Map resolution (Å)  FSC threshold | 2.85  0.143 |
| Map resolution range (Å) | 2.5-4.5 |
| **Refinement** |  |
| Model resolution (Å)  FSC threshold | 3.0  0.5 |
| Model composition  Non-hydrogen atoms  Protein residues/Nucleotide  Ligands | 7,015  835/18  ZN:2 LIG:1 |
| *B* factors (Å2)  Protein  Ligand | 137.8 |
| R.m.s. deviations  Bond lengths (Å)  Bond angles (°) | 0.005  0.684 |
| Validation  MolProbity score  Clashscore  Rotamers outliers (%) | 1.91  3.52  0.00 |
| Ramachandran plot  Favored (%)  Allowed (%)  Disallowed (%) | 96.25  3.75  0.00 |

**Supplemental Table 2. Blood indices of vehicle and DMT207 treated β-thalassemia mice.**

Results are shown as mean ± SD (n = 5, *p < 0.05, statistical analysis was performed with Student's t-test in the GraphPad Prism 10 software).

| **ITEMs** | **Vehicle** | **DMT207** |
| --- | --- | --- |
| **WBC (10^9/L)** | 10.06±0.97 | 8.38±4.06 |
| **RBC (10^12/L)** | 6.99±1.09 | 5.76±0.79 |
| **HGB (g/L)** | 81.6±5.9 | 74.4±4.41 |
| **MCV (fL)** | 44.78±2.07 | 47.1±1.45 |
| **PLT (10^9/L)** | 1007.4±98.8 | 828.0±185.7 |
| **HCT (%)** | 31.52±6.44 | 27.26±4.41 |
| **MCHC (g/L)** | 296.6±4.2 | 296.6±9.37 |
| **RDW (%)** | 22.38±9.78 | 22.2±10.0 |
| **MCH (pg)** | 11.84±1.12 | 12.4±1.52 |
| **MPV (fL)** | 8.74±0.52 | 9.08±0.54 |
| **PCT (%)** | **0.85±0.08** | **0.68±0.08*** |
| **PDW (%)** | 18.36±2.91 | 19.14±2.76 |

**Supplemental Table 3. Data sheet of actively transcribed gene promoter methylation and FPKM.**

**(This data is provided separately in a .xls file)**

**Supplemental Table 4. Gene list of top transcription upregulation (log2FPKM FC> 1) and promoter demethylation (Δβ < -0.3).**

| **Gene** | **Meth_DMSO** | **Meth_DMT207** | **ΔMeth(Δβ)** | **log2FPKM_DMSO** | **log2FPKM_DMT207** | **log2FPKM_FC** |
| --- | --- | --- | --- | --- | --- | --- |
| PDZK1IP1 | 0.7558615 | 0.1542778 | -0.601584 | 2.4925725 | 8.303103 | 5.8105306 |
| ITLN1 | 0.6713256 | 0.2449264 | -0.426399 | 3.092766 | 7.7121917 | 4.6194257 |
| CLC | 0.4499197 | 0.0882789 | -0.361641 | 3.1038307 | 7.4555836 | 4.3517529 |
| HMOX1 | 0.5020975 | 0.1172831 | -0.384814 | 5.1061978 | 9.1365341 | 4.0303363 |
| **HBG1/2** | **0.7939457** | **0.2276947** | **-0.566251** | **13.482797** | **16.675651** | **3.1928541** |
| AKR1C1 | 0.6354415 | 0.1973929 | -0.438049 | 2.6856166 | 5.7525412 | 3.0669246 |
| OSGIN1 | 0.5386966 | 0.1425525 | -0.396144 | 2.4231491 | 5.3921139 | 2.9689648 |
| HBZ | 0.8284082 | 0.2578876 | -0.570521 | 2.7588345 | 5.4686258 | 2.7097912 |
| ATF3 | 0.5828034 | 0.2261909 | -0.356613 | 2.7835311 | 5.2334842 | 2.4499531 |
| SAT1 | 0.5363726 | 0.0769542 | -0.459418 | 4.8606945 | 7.3026588 | 2.4419643 |
| MAP1LC3B2 | 0.5724254 | 0.1513857 | -0.42104 | 2.5462451 | 4.8320151 | 2.28577 |
| SRGN | 0.4493505 | 0.0983729 | -0.350978 | 5.481113 | 7.752947 | 2.2718339 |
| TMEM86B | 0.5809162 | 0.170937 | -0.409979 | 3.2843287 | 5.5493912 | 2.2650625 |
| PLAAT4 | 0.5497522 | 0.1932051 | -0.356547 | 2.6926862 | 4.8429531 | 2.1502669 |
| HBBP1 | 0.8642416 | 0.203854 | -0.660388 | 5.0617535 | 7.1813488 | 2.1195953 |
| TSPO2 | 0.5373983 | 0.1252272 | -0.412171 | 3.7393734 | 5.8403315 | 2.1009581 |
| PINK1 | 0.5891892 | 0.16362 | -0.425569 | 2.7551284 | 4.8455416 | 2.0904132 |
| PRG2 | 0.8273438 | 0.1793979 | -0.647946 | 3.6218836 | 5.6317789 | 2.0098953 |
| HSD17B11 | 0.5152321 | 0.1719782 | -0.343254 | 3.8304728 | 5.7907228 | 1.96025 |
| PLEK2 | 0.5393531 | 0.1961162 | -0.343237 | 2.9914228 | 4.9459937 | 1.9545709 |
| BGLT3 | 0.8079247 | 0.1955123 | -0.612412 | 3.9279572 | 5.8528752 | 1.9249181 |
| UBXN6 | 0.7409112 | 0.1772509 | -0.56366 | 6.0747671 | 7.9658421 | 1.891075 |
| ANXA1 | 0.7825101 | 0.2560161 | -0.526494 | 3.2217541 | 5.0808052 | 1.8590511 |
| GDF15 | 0.5247958 | 0.157632 | -0.367164 | 8.0153558 | 9.8673893 | 1.8520336 |
| TFE3 | 0.7422395 | 0.3085567 | -0.433683 | 2.5052387 | 4.3164564 | 1.8112177 |
| ISG20 | 0.5971592 | 0.1721742 | -0.424985 | 2.5160412 | 4.3201539 | 1.8041127 |
| TMCC2 | 0.523169 | 0.1168431 | -0.406326 | 2.6669252 | 4.4011499 | 1.7342247 |
| BBC3 | 0.4643398 | 0.1337623 | -0.330577 | 4.5887825 | 6.227043 | 1.6382606 |
| KLHDC8B | 0.4317626 | 0.1228708 | -0.308892 | 5.1625703 | 6.7448118 | 1.5822415 |
| RRP12 | 0.5680693 | 0.1938974 | -0.374172 | 2.7078551 | 4.2696689 | 1.5618138 |
| FKBP8 | 0.7691738 | 0.2377837 | -0.53139 | 7.4424895 | 9.0010963 | 1.5586068 |
| PSAP | 0.7121453 | 0.2049861 | -0.507159 | 7.2578179 | 8.7983671 | 1.5405492 |
| TNIP1 | 0.4710824 | 0.1360265 | -0.335056 | 3.9457386 | 5.4850726 | 1.5393339 |
| ARG1 | 0.8375873 | 0.2897803 | -0.547807 | 2.6669255 | 4.1420323 | 1.4751068 |
| CES2 | 0.5530222 | 0.1409806 | -0.412042 | 3.0481697 | 4.5115186 | 1.4633489 |
| ALDH3B1 | 0.4990502 | 0.1576615 | -0.341389 | 3.5335765 | 4.9941511 | 1.4605747 |
| KCNK1 | 0.5978371 | 0.1799105 | -0.417927 | 3.4182633 | 4.8602458 | 1.4419825 |
| BAG1 | 0.4322016 | 0.1055383 | -0.326663 | 4.1991246 | 5.6010541 | 1.4019295 |
| MYDGF | 0.4925854 | 0.1628665 | -0.329719 | 4.1809446 | 5.5679496 | 1.387005 |
| GET4 | 0.7236491 | 0.2886819 | -0.434967 | 4.1647344 | 5.5428694 | 1.378135 |
| YBX3 | 0.5763884 | 0.1530374 | -0.423351 | 6.8632027 | 8.2413343 | 1.3781316 |
| EMID1 | 0.4597369 | 0.1281274 | -0.33161 | 2.7525369 | 4.1124836 | 1.3599467 |
| GTPBP2 | 0.6479553 | 0.1702376 | -0.477718 | 5.3121372 | 6.6720741 | 1.3599369 |
| RMND5A | 0.6747588 | 0.1966056 | -0.478153 | 3.9763517 | 5.3328553 | 1.3565036 |
| RRAGC | 0.6452651 | 0.1941831 | -0.451082 | 4.2603384 | 5.5506136 | 1.2902752 |
| SMOX | 0.5010961 | 0.1551524 | -0.345944 | 4.3995249 | 5.644929 | 1.2454041 |
| VASP | 0.4734435 | 0.130532 | -0.342912 | 2.512522 | 3.7524979 | 1.2399759 |
| AMFR | 0.5172086 | 0.1837731 | -0.333436 | 4.23193 | 5.4534778 | 1.2215478 |
| MCOLN1 | 0.5396641 | 0.1854487 | -0.354215 | 2.812392 | 3.9642298 | 1.1518378 |
| HERPUD1 | 0.4117199 | 0.1067441 | -0.304976 | 3.5411242 | 4.6611608 | 1.1200366 |
| AK1 | 0.6310859 | 0.138009 | -0.493077 | 4.659443 | 5.7719537 | 1.1125106 |
| PIH1D1 | 0.5043774 | 0.1479262 | -0.356451 | 3.6160768 | 4.7231448 | 1.107068 |
| CFDP1 | 0.4755061 | 0.1464321 | -0.329074 | 4.4214614 | 5.5270329 | 1.1055716 |
| LPIN2 | 0.4513508 | 0.1177599 | -0.333591 | 4.7107941 | 5.8113244 | 1.1005303 |
| CA1 | 0.734614 | 0.1948014 | -0.539813 | 8.9104638 | 9.9320302 | 1.0215664 |
| KRCC1 | 0.494329 | 0.1452544 | -0.349075 | 3.2178309 | 4.2348256 | 1.0169947 |
| AHSP | 0.5679353 | 0.0842007 | -0.483735 | 10.387211 | 11.396147 | 1.0089361 |
| GOLGA3 | 0.4782532 | 0.166542 | -0.311711 | 2.7747597 | 3.7813588 | 1.0065991 |

**Supplemental Table 5. Top hits from mass-spectrometry analysis of FLAG-tagged immunoprecipitation from DMT207-treated HUDEP-2 FLAG-DNMT1 stable cell line, compared to vehicle-treated control.**

| **Gene** | **Log2 protein abundance**  **(DMT207/DMSO)** | **p-value** |
| --- | --- | --- |
| SSRP1 | 2.062108254 | 0.003376 |
| JMJD1C | 1.139771338 | 0.003906 |
| SUPT16H | 2.789136289 | 0.004534 |
| **UHRF1** | **1.031959167** | **0.005177** |
| SCML2 | 1.074737159 | 0.005306 |
| H3.1/H3.2 | 0.743535528 | 0.013856 |
| DMAP1 | 0.803844453 | 0.015868 |
| PKM | -0.783431731 | 0.025569 |
| GMPS | 0.887155812 | 0.027399 |
| PCLAF | 1.215038716 | 0.029555 |
| ZNF326 | 0.954708623 | 0.031619 |
| TCF20 | 1.121022038 | 0.039146 |
| TPX2 | 1.019413219 | 0.046123 |
| WDR18 | 2.76354549 | 0.048765 |
| USP7 | 0.50149277 | 0.049558 |

**Supplemental Table 6. Primers used in this study.**

| **Primers** | **Sequences (5'-3')** |
| --- | --- |
| HBA-F | AAGACCTACTTCCCGCACTTC |
| HBA-R | GTTGGGCATGTCGTCCAC |
| HBB-F | TGGGCAACCCTAAGGTGAAG |
| HBB-R | GTGAGCCAGGCCATCACTAAA |
| HBG-F | TGGCAAGAAGGTGCTGACTTC |
| HBG-R | GCAAAGGTGCCCTTGAGATC |
| GATA1-F | CTGTCCCCAATAGTGCTTATGG |
| GATA1-R | GAATAGGCTGCTGAATTGAGGG |
| GAPDH-F | AGCCACATCGCTCAGACAC |
| GAPDH-R | GCCCAATACGACCAAATCC |
| εy-globin-F | TGGCCTGTGGAGTAAGGTC AA |
| εy-globin-R | GAAGCAGAGGACAAGTTCCC A |
| βh1-globin-F | GAAACCCCCGGATTAGAGC C |
| βh1-globin-R | GAGCAAAGGTCTCCTTGAGG T |
| βmaj-globin-F | GGGTAATGCCAAAGTGAAG GC |
| βmaj-globin-R | GGCCCAGCACAATCACGATCA T |
| βmin-globin-F | TCTGCTGTCTCTTGCCTGTG |
| βmin-globin-R | CCTTTTTGCCATGGGCCTTC |
| mActin-F | GGCTGTATTCCCCTCCATCG |
| mActin-R | CCAGTTGGTAACAATGCCATG T |
| sgUHRF1-F | caccgCGACGTGAACAGACTCTGCC |
| sgUHRF1-R | aaacGGCAGAGTCTGTTCACGTCGc |
| sgNEG-F | caccgGCACTACCAGAGCTAACTCA |
| sgNEG-R | aaacTGAGTTAGCTCTGGTAGTGCc |
| DNMT1-cloning-F | AAACGCGGATCCGCGACGCGTCGGTAGCTTGGGGCCACCATGGATTACAAAGACGATGA |
| DNMT1-cloning-R | CCGTAGTTTGAATTCCATATGGAATTCCCTAGTCCTTAGCAGCTTCCTCC |

**Supplemental Figures**

**
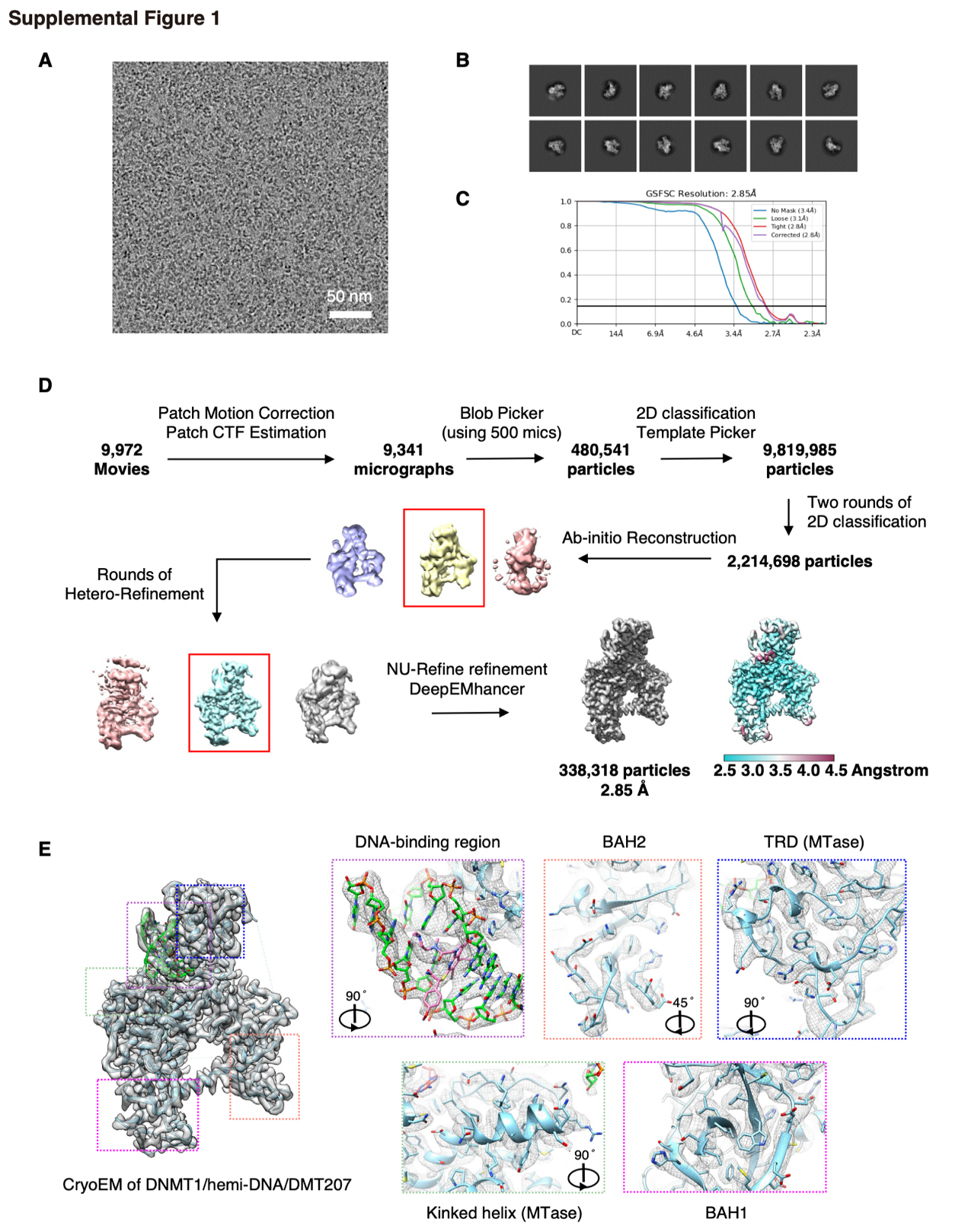
**

**Supplemental figure 1 related to Fig. 1**

**Figure S1. Cryo-EM analysis of the DNMT1/hemi-DNA/DMT207 complex.**

**(A)** Representative cryo-EM micrograph of the DNMT1/hemi-DNA/DMT207 complex. **(B)** Representative 2D averages of the DNMT1/hemi-DNA/DMT207 complex.

**(C)** Fourier shell correlation curves of cryo-EM map for the DNMT1/hemi-DNA/DMT207 complex.

**(D)** Cryo-EM processing flowchart of the DNMT1/hemi-DNA/DMT207 complex.

**(E)** Cryo-EM map and structure in indicated regions of DNMT1/hemi-DNA/DMT207 complex.

**
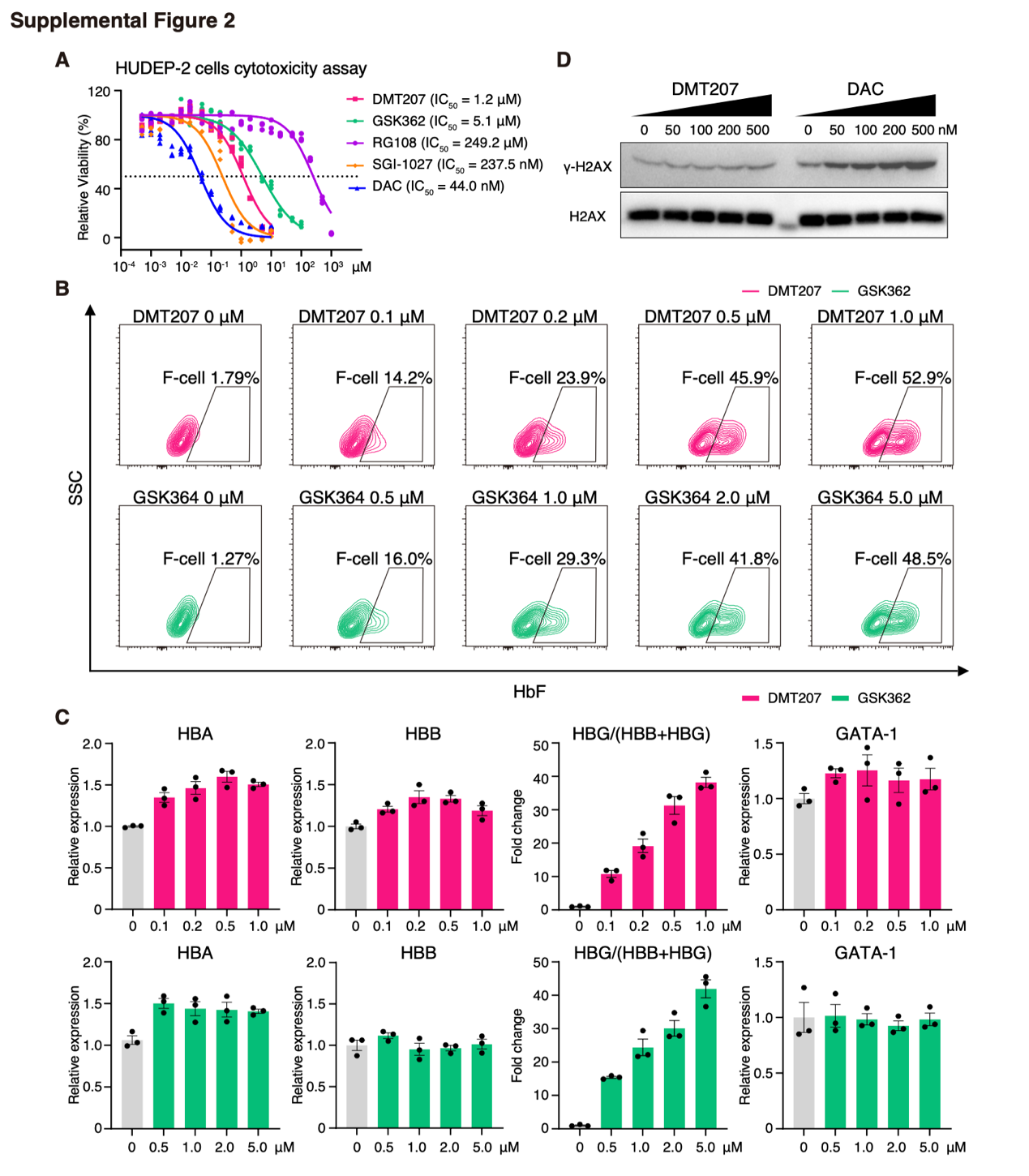
**

**Supplemental figure 2 related to Fig. 1**

**Figure S2. Comparison of DNMT inhibitors in HUDEP-2 cells.**

**(A)** Cytotoxicity assays of DNMT inhibitors on HUDEP-2 cells (n = 4).

**(B)** Flow cytometry analysis of HbF-expressing HUDEP-2 cells after drug treatment.

**(C)** Relative mRNA level of globin genes and *GATA-1* of HUDEP-2 cells (normalized to GAPDH) and the relative ratio of HBG/(HBB+HBG). Results are shown as mean ± SD (n = 3).

**(D)** Western blots of HUDEP-2 cells treated with serial concentrations of DAC or DMT207 for 2 days.

**
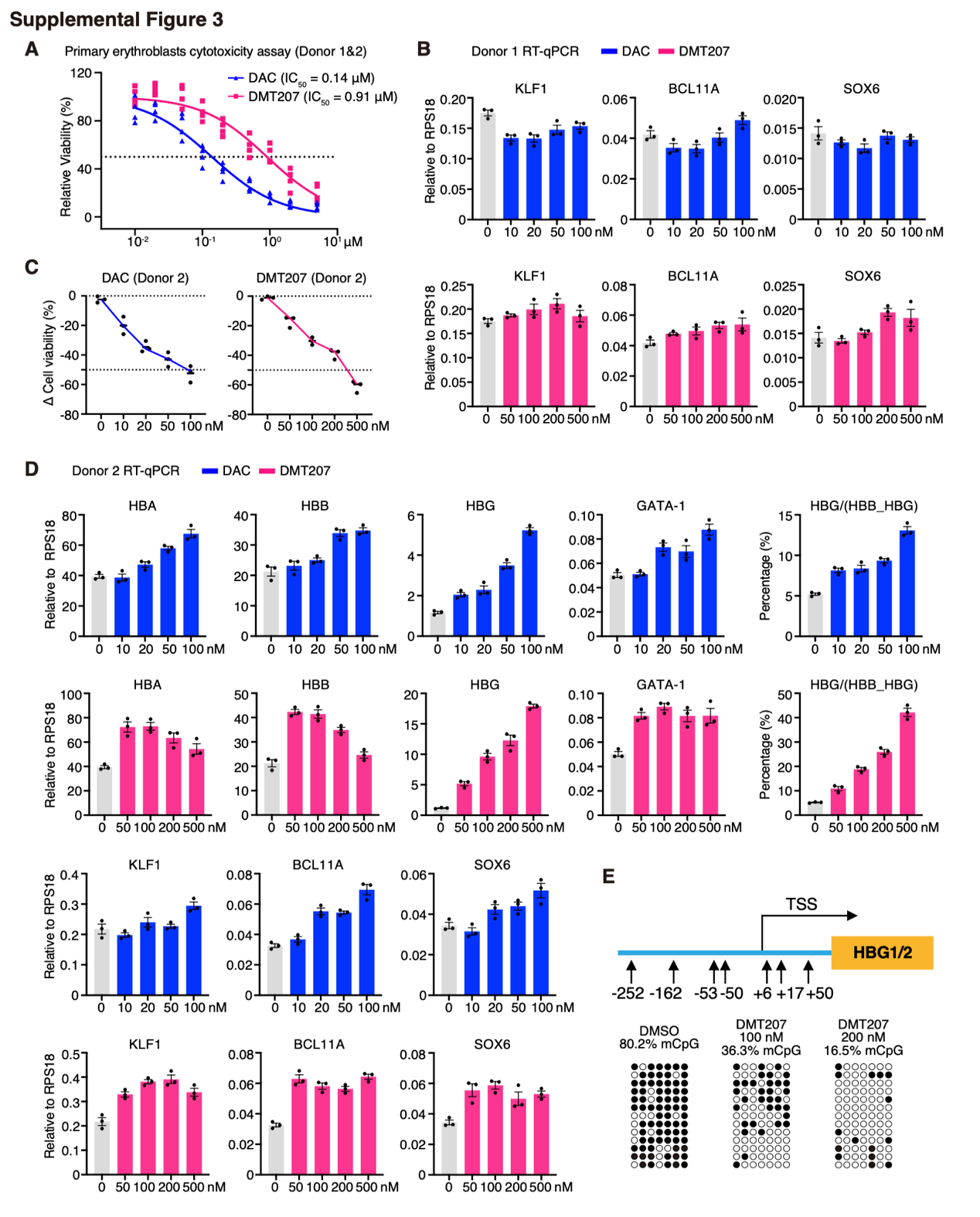
**

**Supplemental figure 3 related to Fig. 2**

**Figure S3. DMT207 treatment induces more HbF than DAC in erythroid cells from Donor 2.**

**(A)** Cytotoxicity assays of DMT207 and DAC on primary erythroblasts from donor 1 and donor 2 (2 donors, for each donor, n = 2).

**(B)** Relative expression of KLF1, BCL11A and SOX6 from donor 1. RPS18 was used for normalization. Results are shown as mean ± SD (n = 3).

**(C)** Δ Cell viability of adult primary erythroblasts on day 16 (donor 2, n=3).

**(D)** Relative expression of globin genes and regulators in adult primary erythroblasts from donor 2, treated with serial concentrations of DAC and DMT207 on day 16. RPS18 was used for normalization. Results are shown as mean ± SD (n = 3).

**(E)** The CpG sites of *HBG* promoter and the mCpG level in adult primary erythroblasts from donor 1 and donor 2, treated with serial concentrations of DMT207 on day 16.

**
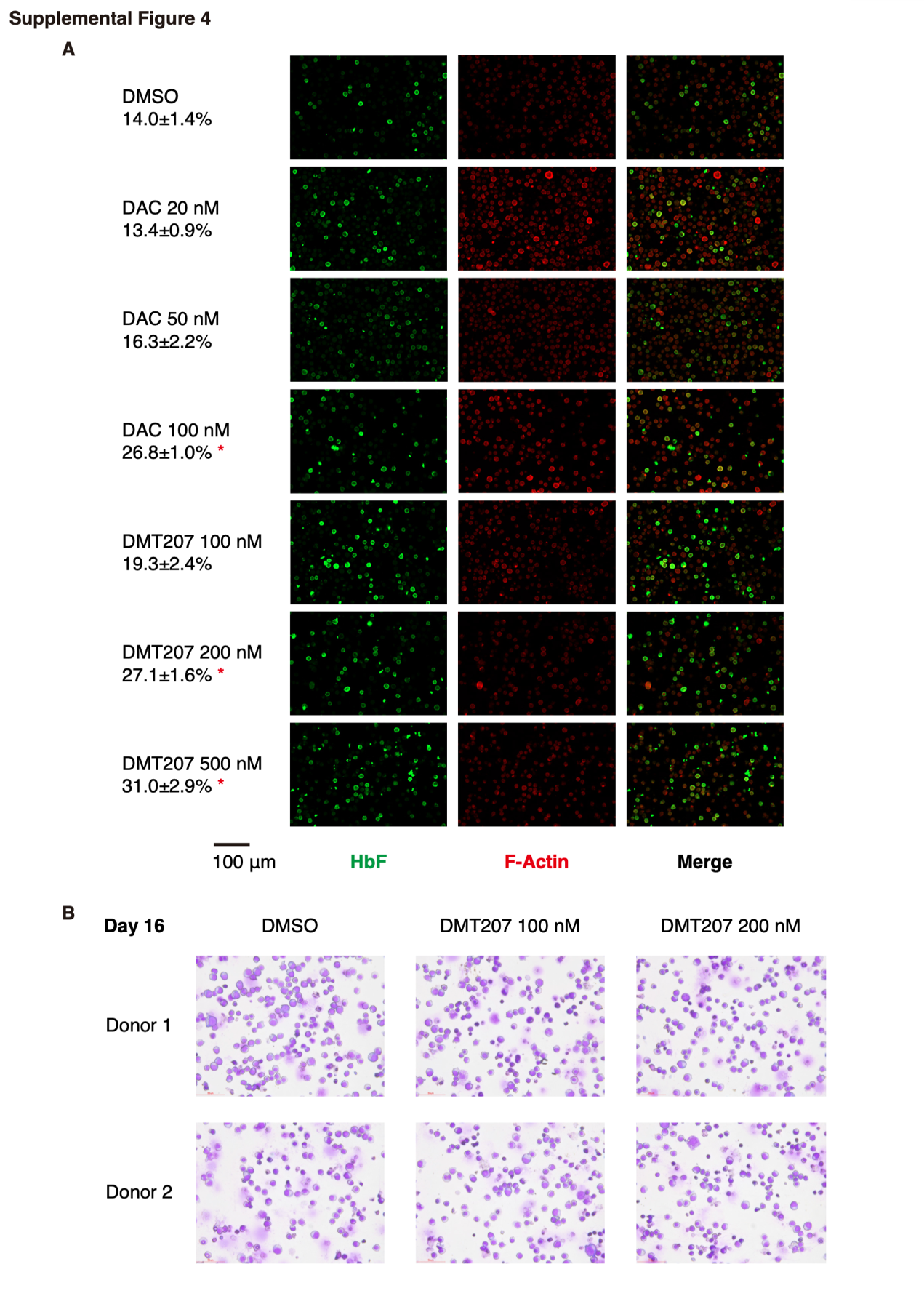
**

**Supplemental figure 4 related to Fig. 2**

**Figure S4. Morphological and quantitative imaging analysis of primary erythroblasts.**

**(A)** Immunofluorescence staining of HbF (green) and F-ACTIN (red) of primary erythroblasts. Strong HbF^+^ cells were analyzed using the ImageJ software. Results are shown as mean ± SD (n = 2, *p < 0.05, statistical analysis was performed with Student's t-test in the GraphPad Prism 10 software).

**(B)** Wright-Giemsa staining of cultured adult primary erythroblasts on day 16.

**
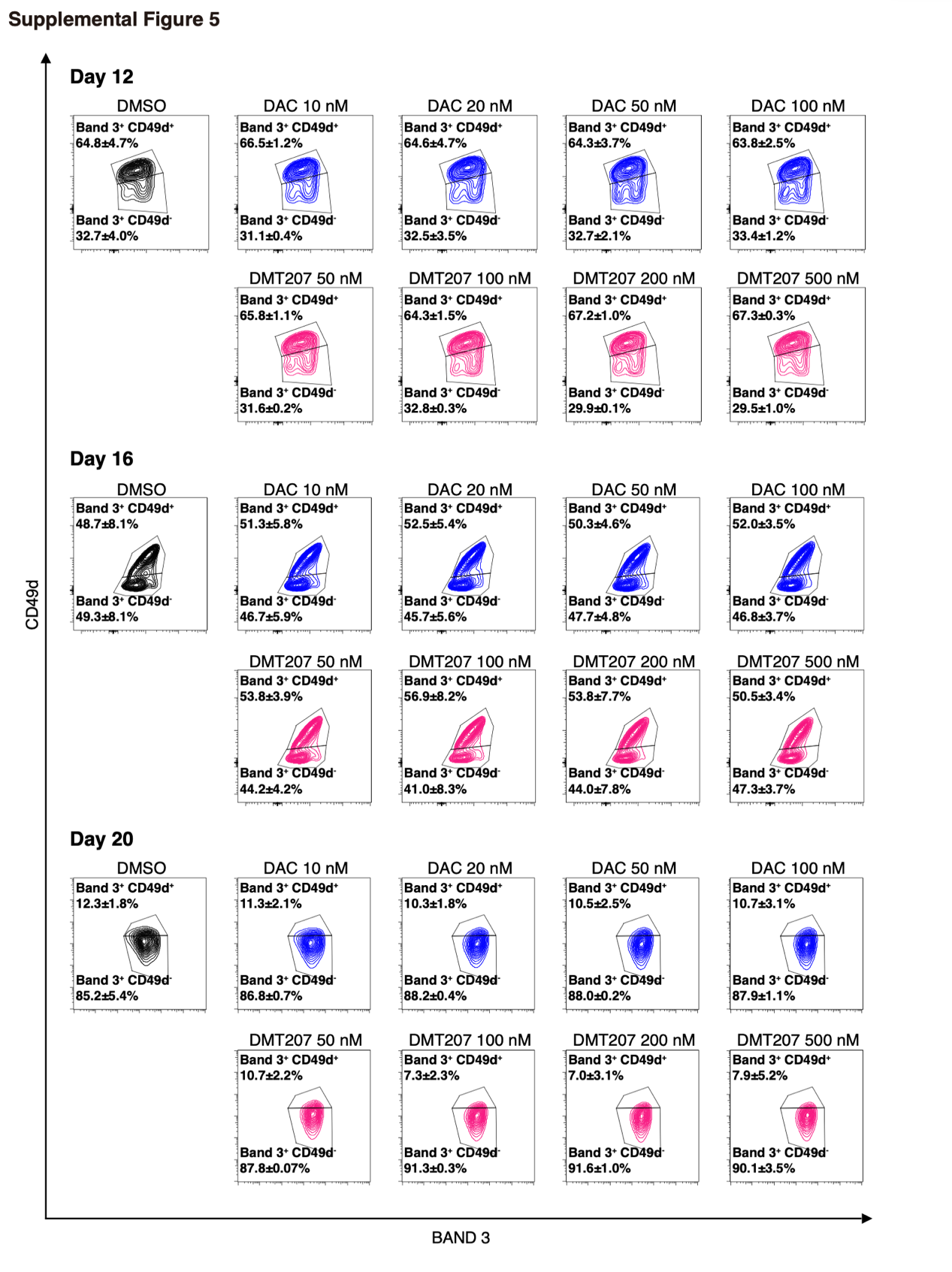
**

**Supplemental figure 5 related to Fig. 2**

**Figure S5. Flow analysis of primary erythroblasts (BAND 3 and CD49d).**

Representative flow analysis of erythroid maturation markers BAND 3 and CD49d in cultured adult primary erythroblasts treated with serial concentrations of decitabine (DAC) and DMT207. Results are shown as mean ± SD (n = 2). No statistical significance was found with t-test using the GraphPad Prism 10 software.

**
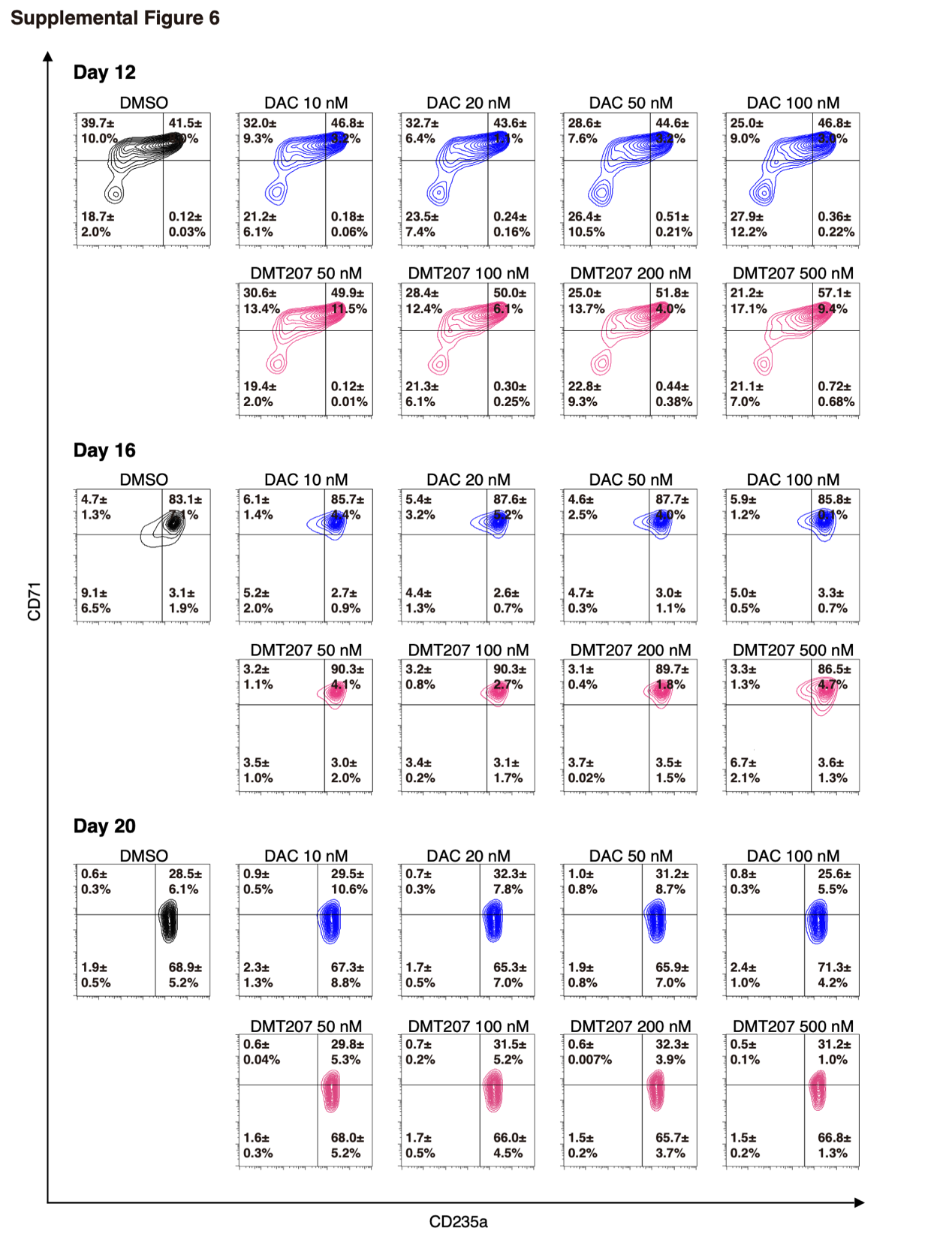
**

**Supplemental figure 6 related to Fig. 2**

**Figure S6. Flow analysis of primary erythroblasts (CD71 and CD235a).**

Representative flow analysis of erythroid maturation markers CD71 and CD235a in cultured adult primary erythroblasts treated with serial concentrations of decitabine (DAC) and DMT207. Results are shown as mean ± SD (n = 2). No statistical significance was found with t-test using the GraphPad Prism 10 software.

**
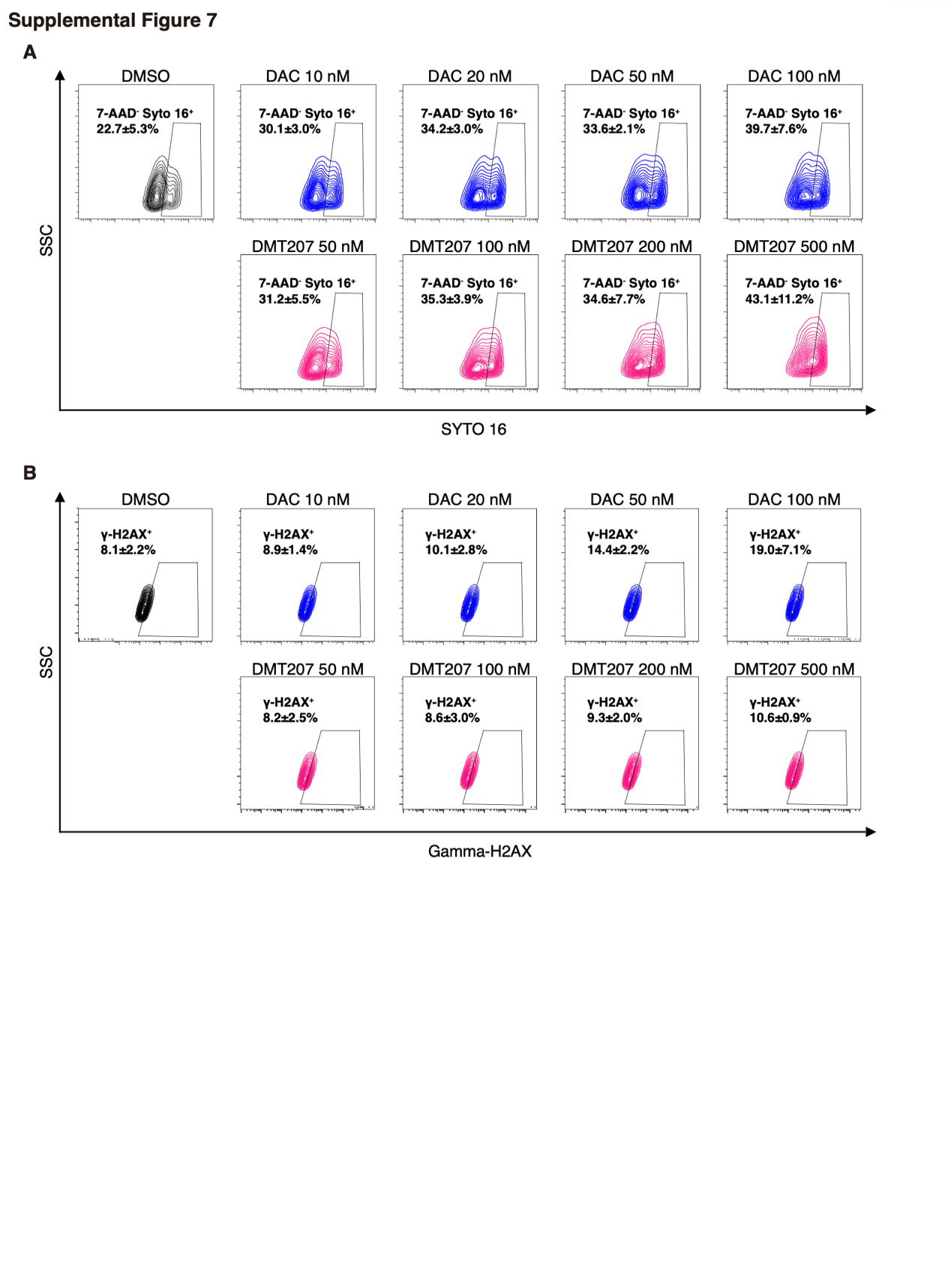
**

**Supplemental figure 7 related to Fig. 2**

**Figure S7. Flow analysis of primary erythroblasts (enucleation and γ-H2AX).**

**(A)** Representative flow analysis of erythroid enucleation using Syto 16 and 7-AAD in cultured adult primary erythroblasts treated with serial concentrations of decitabine (DAC) and DMT207. Results are shown as mean ± SD (n = 2). No statistical significance was found with t-test using the GraphPad Prism 10 software.

**(B)** Representative flow analysis of γ-H2AX in cultured adult primary erythroblasts treated with serial concentrations of decitabine (DAC) and DMT207. Results are shown as mean ± SD (n = 2).

**
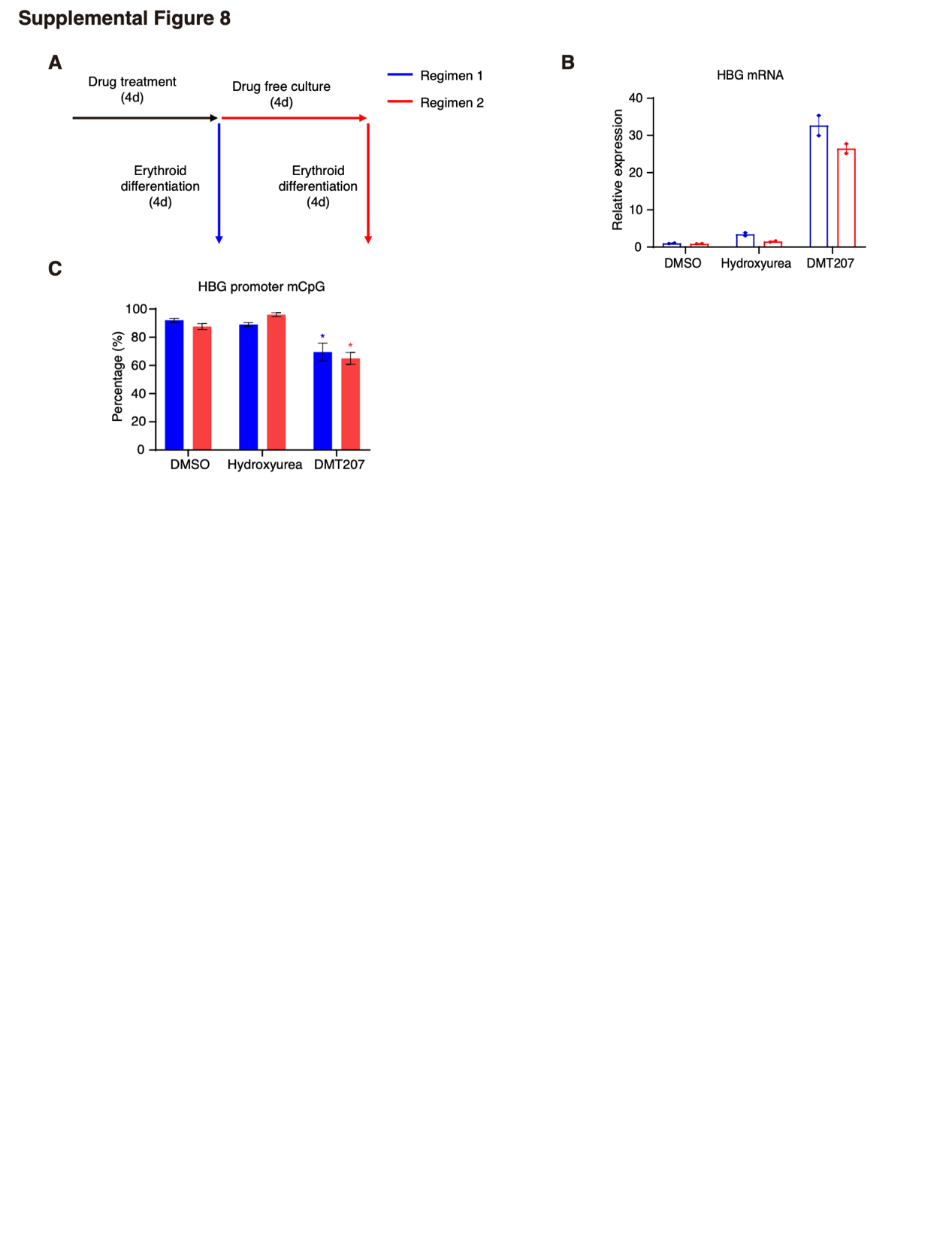
**

**Supplemental figure 8 related to Fig. 3**

**Figure S8. DMT207 transient treatment activates *HBG* expression.**

**(A)** Treatment regimen schematic for transient exposure to compounds. HUDEP-2 cells were treated with either DMSO, DMT207 (200 nM), or hydroxyurea (Hu, 50 µM) for 4 days in the expansion medium. For regimen 1, HUDEP-2 cells were induced for differentiation immediately following treatment. For regimen 2, HUDEP-2 cells were grown in the expansion medium for an additional 4 days (compounds washed out), followed by differentiation induction.

**(B)** The relative mRNA level of *HBG* in HUDEP-2 cells in 2 regimens (normalized to ACTB). Results are shown as mean ± SD (n = 2).

**(C)** mCpG levels of *HBG* promoters in HUDEP-2 cells in 2 regimens. Results are shown as mean ± SD (n = 2, *p < 0.05, statistical analysis was performed with Student's t-test in the GraphPad Prism 10 software).

**
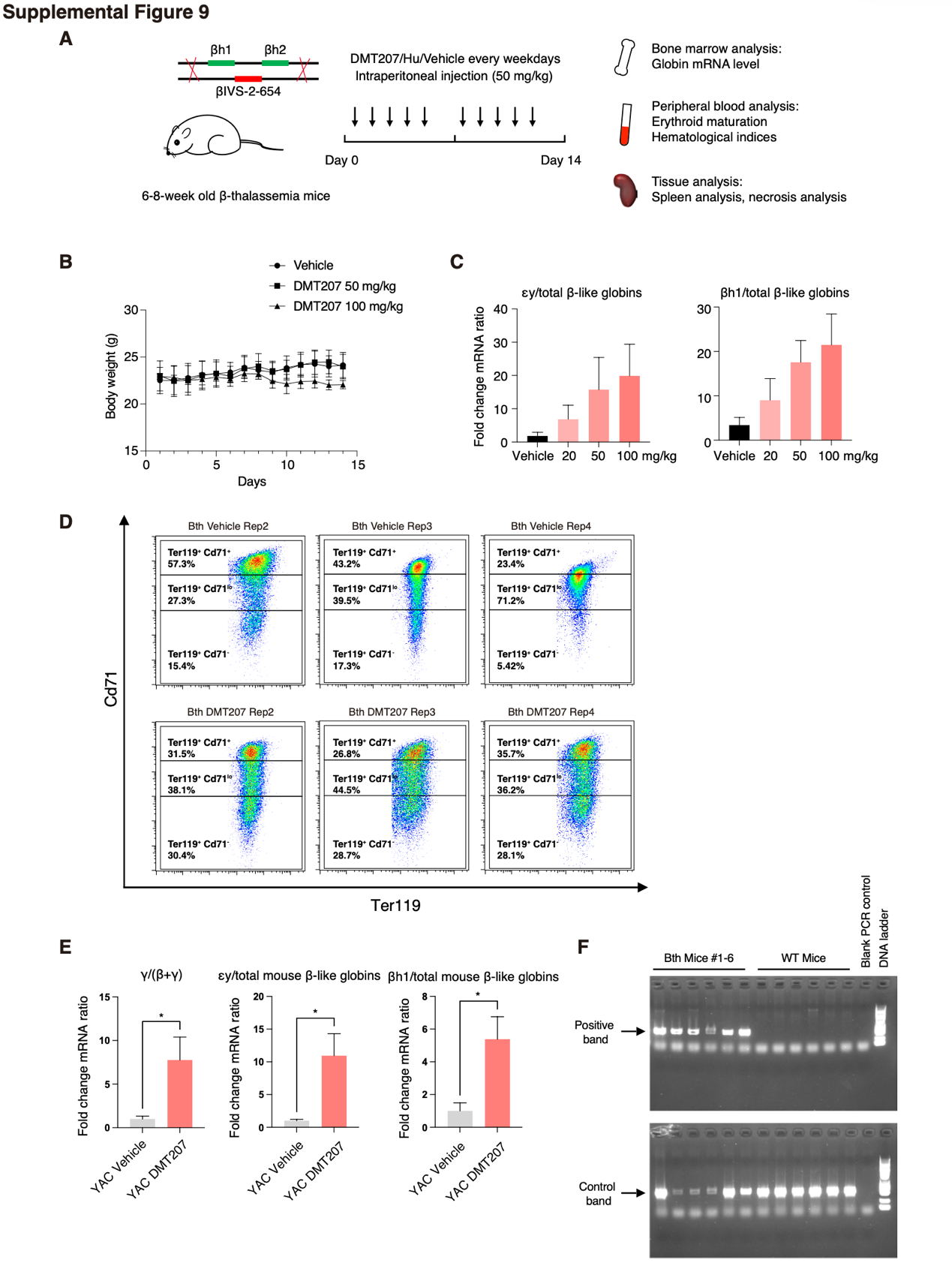
**

**Supplemental figure 9 Related to Fig. 4**

**Figure S9. DMT207 induces γ-globin in β-YAC mice.**

**(A)** *In vivo* study design of DMT207. Six-eight-week-old β-thalassemia mice were grouped into vehicle, DMT207 and hydroxyurea (Hu) treated groups (n = 5). Mice were administered vehicle or DMT207 or Hu via intraperitoneal injection on weekdays. On day 14, mice were sacrificed. Peripheral blood, bone marrow, spleen and liver were collected for downstream analysis.

**(B)** Mouse body weight of vehicle and DMT207 (50 and 100 mg/kg) treated groups. Results are shown as mean ± SD (n = 5).

**(C)** Fold change mRNA ratio of εy- and βh1-globin to total β-like globin (normalized to mActin). Results are shown as mean ± SD (n = 5).

**(D)** Representative flow analysis of peripheral blood erythroid cells stained with cell differentiation markers CD71 and Ter119.

**(E)** Fold change mRNA ratio of γ-globin to human total β-like globin and εy- and βh1-globin to murine total β-like globin in β-YAC mice (normalized to mActin). Results are shown as mean ± SD (n = 4, *P < 0.05, statistical analysis was performed with Student's t-test in the GraphPad Prism 10 software).

**(F)** Representative gel graphs of Bth mice genotyping.

**
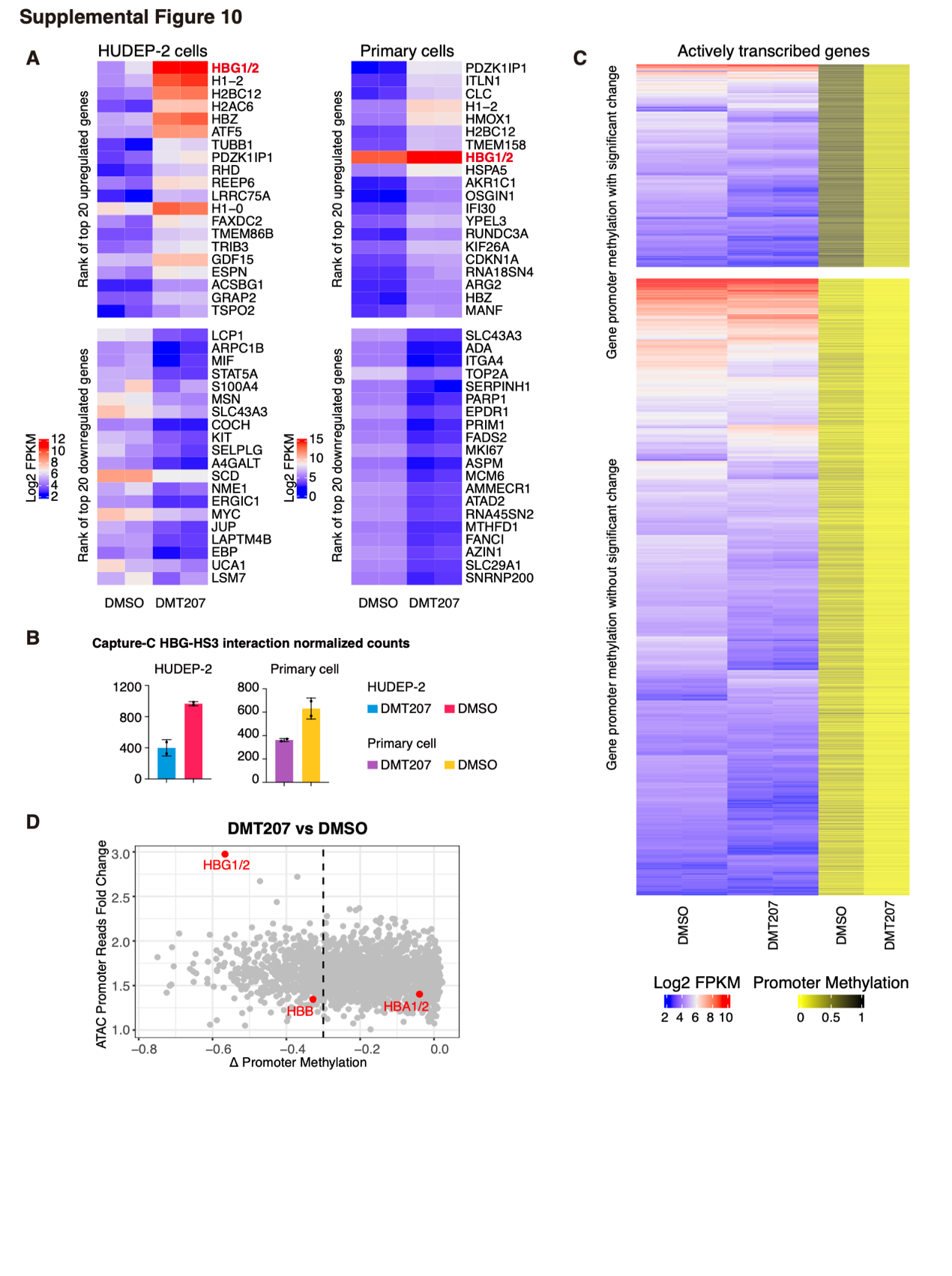
**

**Supplemental figure 10 related to Fig. 5**

**Figure S10. Multi-omics studies of global effects of DMT207.**

**(A)** Top 20 of differentially expressed genes from RNA-seq analysis (DMT207 vs DMSO).

**(B)** Normalized Capture-C read counts of chromatin HBG-HS3 interaction. Results are shown as mean ± SD (n = 2).

**(C)** Heatmap of gene transcription log_2_(FPKM) and promoter methylation of actively transcribed genes, clustered as promoter methylation non-significantly changed (Δβ ≥ -0.3) and significantly changed (Δβ < -0.3).

**(D)** Scatter plot of promoter ATAC-seq reads fold change and promoter Δ methylation of actively transcribed genes (DMT207 vs DMSO).

**
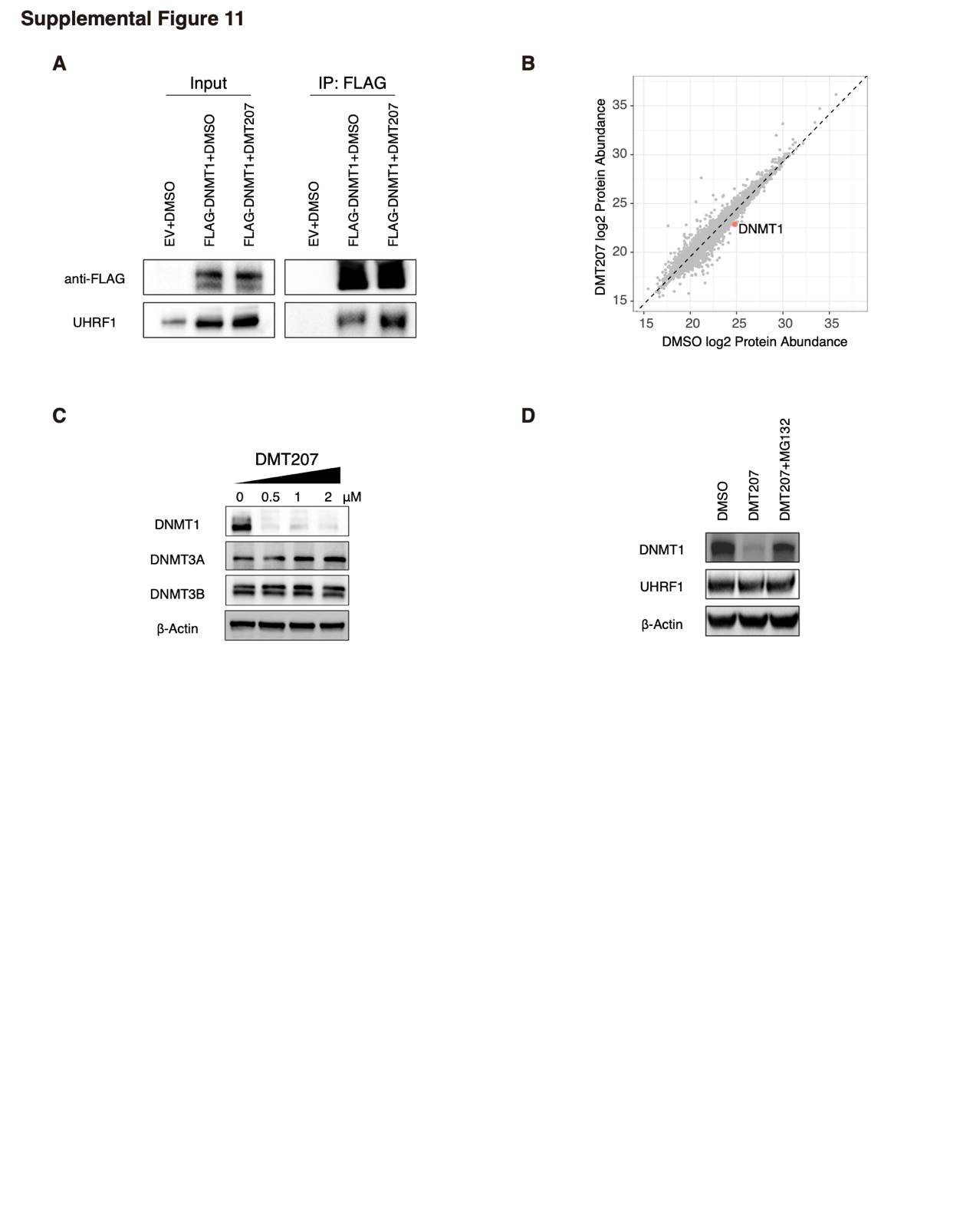
**

**Supplemental figure 11 Related to Fig. 6**

**Figure S11. DMT207 affects the binding of DNMT1 to its partner in 293T cells.**

**(A)** FLAG-tagged IP-WB of HEK293T cell lysates transfected with FLAG-DNMT1 and treated with vehicle or DMT207 for 2d.

**(B)** Scatter plot of mass-spectrometry analysis in adult primary erythroblasts (DMT207 vs DMSO). DNMT1 are highlighted.

**(C)** Western blots of K562 cells treated with DMT207 for 2d.

**(D)** Western blots of HUDEP-2 cells treated with DMSO, DMT207, and DMT207 + MG132.
